# Mesencephalic dopaminergic neurons express a repertoire of olfactory receptors and respond to odorant-like molecules

**DOI:** 10.1186/1471-2164-15-729

**Published:** 2014-08-27

**Authors:** Alice Grison, Silvia Zucchelli, Alice Urzì, Ilaria Zamparo, Dejan Lazarevic, Giovanni Pascarella, Paola Roncaglia, Alejandro Giorgetti, Paula Garcia-Esparcia, Christina Vlachouli, Roberto Simone, Francesca Persichetti, Alistair RR Forrest, Yoshihide Hayashizaki, Paolo Carloni, Isidro Ferrer, Claudia Lodovichi, Charles Plessy, the FANTOM Consortium, Piero Carninci, Stefano Gustincich

**Affiliations:** SISSA, Area of Neuroscience, via Bonomea 265, 34136 Trieste, Italy; Department of Health Sciences, University of Eastern Piedmont “A. Avogadro”, via Solaroli 17, 28100 Novara, Italy; Venetian Institute of Molecular Medicine (VIMM), via Orus 2, 35129 Padua, Italy; CBM, AREA Science Park, s.s. 14, Km 163.5, Basovizza, 34012 Trieste Italy; RIKEN Omics Science Center, Yokohama, Kanagawa 230-0045 Japan; RIKEN Center for Life Science Technologies, Division of Genomic Technologies, Yokohama Institute, 1-7-22 Suehiro-cho, Tsurumi-ku, Yokohama, Kanagawa 230-0045 Japan; Computational Biophysics, German Research School for Simulation Sciences (Joint venture of RWTH Aachen University and Forschungszentrum Jülich), Jülich, Germany; Department of Biotechnology, University of Verona, Ca’ Vignal 1, Strada Le Grazie 15, 37134 Verona, Italy; IDIBELL-University Hospital of Bellvitge, Institute of Neuropathology, Carrer Feixa Llarga sn, 08907 Hospitalet de Llobregat, Spain; RIKEN Preventive Medicine and Diagnosis Innovation Program, Wako, Saitama 351-0198 Japan; Institute for Advanced Simulation IAS-5, Computational Biomedicine, Forschungszentrum Jülich, Jülich, Germany; Computational Biomedicine Section INM-9, Institute for Neuroscience and Medicine, Jülich, Germany; The Giovanni Armenise-Harvard Foundation Laboratory, SISSA, via Bonomea 265, 34136 Trieste, Italy; European Molecular Biology Laboratory, European Bioinformatics Institute (EMBL-EBI), Wellcome Trust Genome Campus, Hixton, Cambridge CB10 1SD UK

**Keywords:** NanoCAGE, Odors, Odorant receptors, Dopaminergic neurons, Ventral midbrain

## Abstract

**Background:**

The mesencephalic dopaminergic (mDA) cell system is composed of two major groups of projecting cells in the Substantia Nigra (SN) (A9 neurons) and the Ventral Tegmental Area (VTA) (A10 cells). Selective degeneration of A9 neurons occurs in Parkinson’s disease (PD) while abnormal function of A10 cells has been linked to schizophrenia, attention deficit and addiction. The molecular basis that underlies selective vulnerability of A9 and A10 neurons is presently unknown.

**Results:**

By taking advantage of transgenic labeling, laser capture microdissection coupled to nano Cap-Analysis of Gene Expression (nanoCAGE) technology on isolated A9 and A10 cells, we found that a subset of Olfactory Receptors (OR)s is expressed in mDA neurons. Gene expression analysis was integrated with the FANTOM5 Helicos CAGE sequencing datasets, showing the presence of these ORs in selected tissues and brain areas outside of the olfactory epithelium. OR expression in the mesencephalon was validated by RT-PCR and *in situ* hybridization. By screening 16 potential ligands on 5 mDA ORs recombinantly expressed in an heterologous *in vitro* system, we identified carvone enantiomers as agonists at *Olfr287* and able to evoke an intracellular Ca^2+^ increase in solitary mDA neurons. ORs were found expressed in human SN and down-regulated in PD *post mortem* brains.

**Conclusions:**

Our study indicates that mDA neurons express ORs and respond to odor-like molecules providing new opportunities for pharmacological intervention in disease.

**Electronic supplementary material:**

The online version of this article (doi:10.1186/1471-2164-15-729) contains supplementary material, which is available to authorized users.

## Background

Dopaminergic (DA) neurons are an anatomically and functionally heterogeneous group of cells involved in a wide range of neuronal network activities and behavior [1]. Among them, mesencephalic dopaminergic neurons (mDA) are the major source of dopamine in the brain. They present two major groups of projecting cells: the A9 neurons of the Substantia Nigra (SN) that form the mesostriatal system and the A10 cells of the Ventral Tegmental Area (VTA) that constitute the mesocorticolimbic pathway [2]. SN neurons are involved in regulating voluntary movements and postural reflexes while VTA cells play a fundamental role in reward and attention.

Dysfunction of DA neurons has been implicated in several neurodegenerative and psychiatric disorders. Selective degeneration of A9 cells leads to Parkinson’s Disease (PD) [3], while altered function of A10 cells has been linked to schizophrenia, attention deficit disorder and addiction [4].

These cells share many characteristics including the enzymatic pathways involved in dopamine synthesis, release and metabolism. They also present common intrinsic electrophysiological properties like a spontaneous pacemaker activity when in absence of synaptic inputs.

The description of the repertoire of genes expressed in mDA neurons may provide crucial information on their physiology and on the mechanisms of cell-type specific dysfunction [5–7].

Cap Analysis of Gene Expression (CAGE) technology was previously developed for the systematic analysis of Transcription Start Sites (TSS)s in eukaryotic cells and tissues [8]. It is based on sequencing cDNA copies of the 5′ends of mRNAs, of which the integrity is inferred by the presence of their cap. These sequences—referred to as *tags*—are sufficiently long to be aligned in most cases at a single location in the genome. The first position of this alignment identifies a base pair where transcription is initiated defining a TSS. The number of times a given tag is represented in a library gives an estimate of the expression level of the corresponding transcript. Our previous analysis with CAGE has shown that promoters can vary in shape, with some genes having a strong preference for initiating transcription from a single genomic position (sharp promoters), while others use a broad collection of TSSs within a region of approximately one hundred bases [9, 10].

In the current FANTOM5 project, a modified protocol of CAGE for high-throughput single molecule next-generation sequencing with Helicos (hCAGE) has been applied to a wide range of human and mouse tissues providing an unprecedented dataset for promoter usage analysis [11]. Although very broad, the study was limited to samples where 1-5 μg of total RNA could be obtained. To expand this analysis to tiny amounts of *ex vivo* tissue and to the polyA^-^ fraction of RNAs, we developed nanoCAGE, a technology that miniaturizes the requirement of CAGE for RNA material to the nanogram range and which can be used on fixed tissues [12].

NanoCAGE has been recently applied to identify the genome-wide collection of active TSSs of the mouse Olfactory Epithelium (OE) [13]. In this tissue the detection of a vast repertoire of volatile compounds (odors) is accomplished by a large family of Olfactory Receptors (ORs), with more than 1100 intact genes in mouse and about 350 in human. NanoCAGE revealed the map and architecture of promoters for 87.5% of the mouse OR genes [13].

To gain further insights into the physiology and dysfunction of mDA neurons, we have carried out laser capture microdissection (LCM) combined with nanoCAGE technology to profile the genes expressed in A9 and A10 DA cells.

Here we show that a repertoire of OR genes is expressed in mDA neurons (mDA-ORs). We then demonstrate that selected odor molecules stimulate recombinantly expressed mDA-ORs in heterologous cells and trigger Ca^2+^ signaling in isolated primary mDA neurons. Finally, we identify several ORs that are expressed in the human SN and down-regulated in PD.

This work is part of the FANTOM5 project. Data downloads, genomic tools and co-published manuscripts are summarized here: http://fantom.gsc.riken.jp/5/.

## Results

### Identification of ORs transcripts by expression analysis of mDA neurons

We have determined the gene expression profiles of mDA neurons with nanoCAGE technology. To this purpose we took advantage of transgenic mice that selectively express green fluorescent protein (GFP) in catecholaminergic cells under the control of tyrosine hydroxylase (TH) gene promoter (TH-GFP mice) [14]. In this mouse line we can identify the majority of mDA neurons for their GFP labeling. Furthermore, we can distinguish A9 neurons from A10 for their anatomical localization. Thus, LCM and pressure catapulting were used to harvest A9 and A10 cells after fixation with a zinc fix-based method that assured the preservation of both tissue morphology and RNA integrity. RNA was then used as template for nanoCAGE library synthesis. The complete description of A9 and A10-specific transcriptional landscape is presented elsewhere (Lazarevic D, Bertin N, Franke V, Vlachouli C, Caiazzo M, Plessy C, Akalin A, Vatta P, Simone R, Roncaglia P, Daub CO, Faulkner GJ, Broccoli V, Lenhard B, Carninci P, Gustincich S: **The promotorome of adult dopaminergic neurons of the mouse Substantia Nigra identifies new gene networks for cell conversion**. *Submitted*).

These results have been integrated with Affymetrix-based gene expression datasets of A9 and A10 cells available in the laboratory [15].

**Figure 1:**
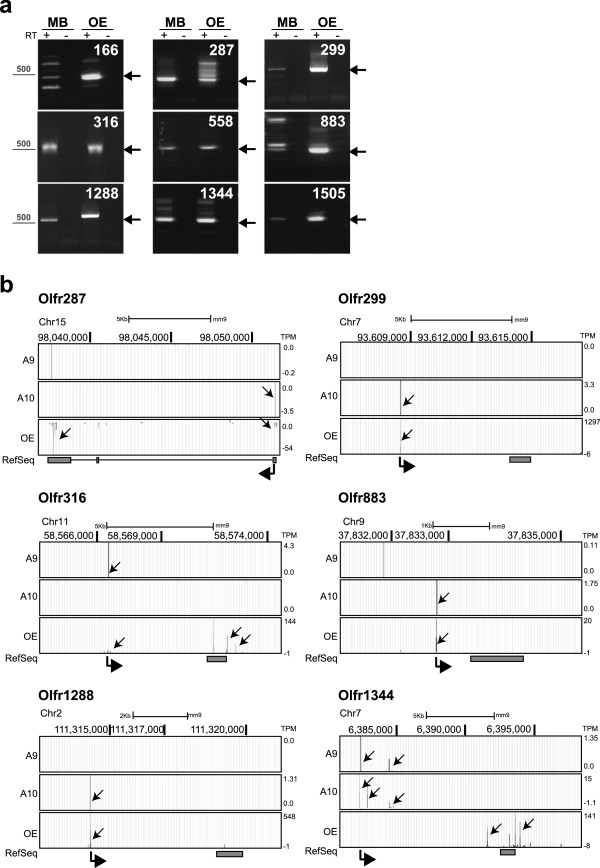
**NanoCAGE analyses unveil atypical expression of OR genes. a)** Validation of OR gene expression in mouse midbrain. MB was dissected from C57Bl/6 mouse and total RNA extracted and used for non-quantitative PCR. Primers were designed to specifically amplify selected ORs, as shown. RNA from OE was included as a positive control. Non-retrotranscribed (-RT) RNA was used as a negative control. Arrows indicate OR specific bands. Data are representative of n = 3 replicas from independent tissue preparations. **b)** Representative tracks of nanoCAGE sequencing data of libraries from A9 and A10 mDA neurons. Data from OE (Plessy et. al., Genome Research, [13]) are included for comparison. Genomic coordinates are shown on top and expression values (TPM) on the right. Positive and negative TPM values indicate transcription on plus and minus DNA filament, respectively. Black arrows in each track highlight TSS. At the bottom, annotated RefSeq is shown in grey and thick arrowheads indicate direction of transcription.

Surprisingly, transcripts for OR genes have been found expressed in mDA neurons (Additional file 1: Table S1). Nine ORs were validated with RT-PCR from ventral midbrain (MB) with the appropriate controls (Figure 1a). Cloning and sequencing of PCR products verified their identity.

5 out of 6 ORs identified by nanoCAGE and validated by RT-PCR display in mDA neurons a TSS very similar if not identical to the canonical TSS found in OE [13] (Figure  1b). OR promoters in mDA cells were of a sharp type, with a single dominant TSS, as in the OE. Expression values, measured as number of tags per million (TPM), ranged from 4.29 (*Olfr316*) to 1.3 (*Olfr1288*).

Since in the OE the functional activation of ORs requires *Gα*_*olf*_ and Adenyl Cyclase III (*Adcy3*), we monitored their expression in our datasets showing that both signaling molecules were present in A9 and A10 mDA neurons (Additional file 2: Figure S1a). Their expression was also detected in FANTOM5 libraries derived from mouse SN (Additional file 2: Figure S1b).

Altogether our results indicate that OR genes as well as components of the olfactory signaling system are expressed in mDA neurons.

### Validation of mDA-OR expression in mDA cells and mouse brain

**Figure 2:**
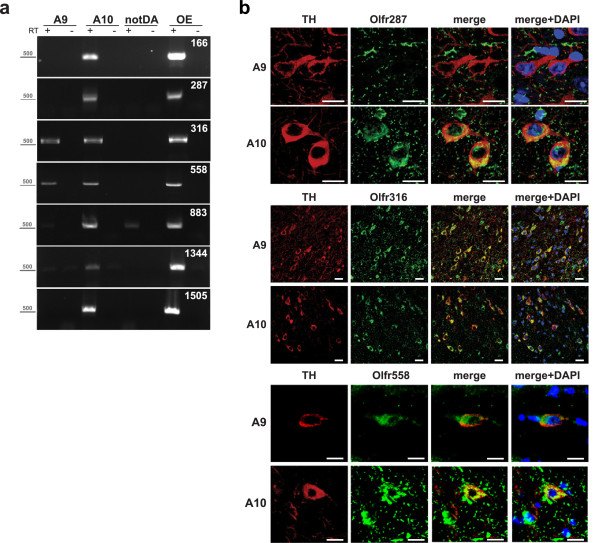
**mDA-ORs are expressed in mDA neurons and in mouse brain. a)** OR genes are expressed in A9 and A10 DA neurons. 2000 DA cells were harvested by LCM. Equal numbers of non-DA cells were collected from the same brain region. OE was also included. Non-quantitative PCR was performed with specific primers. Results are representative of n = 3 independent harvesting. **b)** *In situ* hybridization of selected mDA-ORs in mouse DA neurons. Ventral midbrain slices were processed with *Olfr287*, *Olfr316* and *Olfr558* specific probes (green). DA neurons were visualized by immunohistochemistry with anti-TH antibody (red). Nuclei are in blue (DAPI). Representative images of staining in A9 and A10 neurons are shown (n = 3). Scale bars indicate 20 μm.

RT-PCR and *in situ* hybridization experiments were then carried out to assess mDA-ORs’ cellular distribution. 2000 A9 and A10 DA neurons were harvested with LCM in three independent experiments. As shown in Figure 2a, expression of seven ORs was confirmed in isolated neurons. *Olfr166*, *Olfr287*, *Olfr883*, *Olfr1344* and *Olfr1505* were found exclusively in A10 cells, whereas *Olfr316* and *Olfr558* were present both in A9 and in A10. For *in situ* hybridization (ISH) sense (negative control) and antisense riboprobes were generated for *Olfr287*, *Olfr316* and *Olfr558* and used in a double fluorescent experiment with anti-TH immunoreactivity to identify DA cells [15, 16] (Figure 2b). No or low background staining was measured when sense probes were used (Additional file 3: Figure S2). *Olfr287* was present exclusively in A10 neurons, decorating a portion of TH-positive cells in this area. *Olfr316* and *Olfr558* were expressed in the large majority of A9 and A10 neurons. Altogether, ISH experiments confirmed RT-PCR data on isolated mDA cells.

We then took advantage of an antibody targeting human OR51E1 that we predicted should recognize a group of mouse *Olfrs* including *Olfr558*, the mouse homologue of OR51E1. First, we showed that this antibody was able to specifically stain *Olfr558*-expressing cells upon full-length cDNA transfection in HEK cells (Additional file 4: Figure S3a). We then carried out immunostaining on brain sections, showing that it was decorating all A9 and A10 neurons, plus non-DA cells in the same region as well in the cortex (Additional file 4: Figure S3b). This result proved that OR proteins were present in MB. No signal was detected when only the secondary antibody was used (data not shown).

Overall these data indicate that a subset of ORs is expressed in mDA neurons with an A9/A10 anatomical distribution that is specific for each receptor.

### mDA-ORs are expressed in mouse tissues and cells

**Figure 3:**
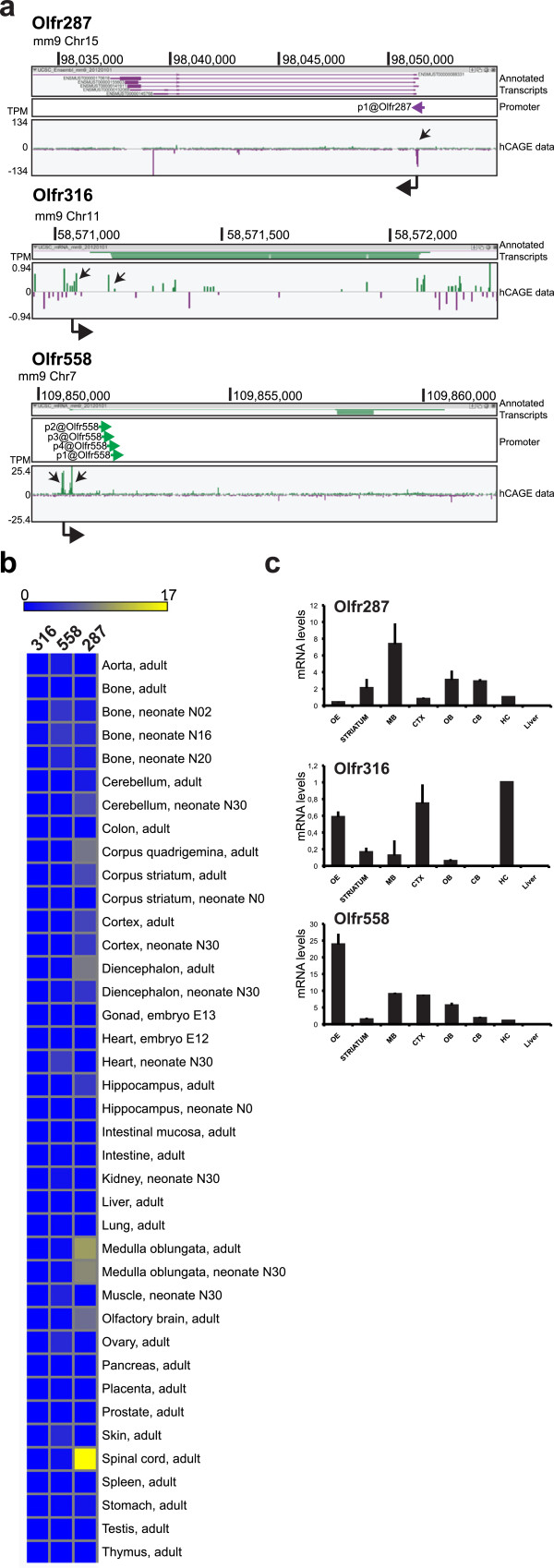
**Expression of mouse mDA-ORs in tissues and primary cells. a)** ZENBU browser view of *Olfr287*, *Olfr316* and *Olfr55*8 TSS in pooled mouse tissues. Genomic coordinates are shown on top and scale of expression values (TPM) on the left. Positive and negative TPM values indicate transcription on plus and minus DNA filament, respectively. Black arrows in each track highlight TSS. At the bottom, thick arrowheads indicate direction of transcription. **b)** FANTOM5 analysis of mDA-OR expression in mouse tissues and various brain regions. **c)** qRT-PCR of OR transcripts expressed in various brain regions. Normalized mRNA levels (ΔΔCt) of *Olfr287*, *Olfr316* and *Olfr558* in mouse tissues. Hippocampus was used as reference and set to 1. OE, olfactory epithelium; MB, ventral midbrain; CTX, cortex; OB, olfactory bulb; CB, cerebellum; HC, hippocampus. Data indicate mean ± st dev and are calculated on three independent experiments.

To assess how widespread is the expression of mDA-ORs (*Olfr316*, *Olfr287* and *Olfr558*) we examined their expression patterns in the FANTOM5 collection of hCAGE mouse libraries (N = 395 datasets). We monitored mDA-OR expression assessing tag counts across the whole locus of interest (sum5end). Expression data was represented as tag per million (TPM). We used the decomposition-based peak identification (DPI) method to identify peaks in CAGE profiles, taking advantage of the deepness of sequencing and the high number of libraries [11]. TSS mapped at almost identical positions confirming the reference sequence annotation (Figure 3a). We detected expression of *Olfr316* in bone, heart, placenta and hippocampus (TPM ranging from 0.7 to 0.1). *Olfr287* was expressed in non-neuronal tissues such as bone (2.52 TPM) and stomach (1.07 TPM) as well as in neuronal tissues such as cerebellum (TPM 1.71-4.97), striatum (TPM 0.16-4.87), cortex (TPM 3.96-4.6), medulla oblongata (TPM 9.16-10.65) and spinal cord (TMP 17.08). *Olfr558* was present in spinal cord (1.02 TPM), medulla oblongata (0.79 TPM), cortex (0.23 TPM) and cerebellum (0.37 TPM) (Figure 3b). No expression was detected in liver, lung, thymus, intestine and testis for any of the mDA-ORs analyzed.

To validate the expression of *Olfr316*, *Olfr287* an *Olfr558*, we dissected several brain regions of adult C57Bl/6 mice and carried out qRT-PCR. OE was included as positive control, while liver was representative of a tissue outside of the central nervous system. All three mDA-ORs showed a distinct pattern in the brain (Figure 3c). Liver scored as negative for all three receptors. *Olfr287* was strongly enriched in MB as compared to the other regions, while *Olfr316* transcript was highest in the hippocampus. *Olfr558* was the only OR with the highest expression in OE.

### Recombinant mDA-ORs are activated by a subset of odors

To investigate the function of mDA-ORs in DA cells’ physiology, we first sought to identify potential agonists for each receptor. Full-length mDA-ORs were cloned from the ventral midbrain for *Olfr166*, *Olfr287*, *Olfr316*, *Olfr558* and *Olfr1344* in frame with a Rho-tag sequence at the N-terminus. cDNAs for the same receptors were also cloned from OE and no sequence differences were observed. Transient transfections in non-neuronal HEK 293 and in dopaminergic iMN9D cells indicated that *Olfr287*, *Olfr316* and *Olfr1344* cloned from MB drove the expression of a protein with the expected molecular weight (Additional file 5: Figure S4a) and whose localization was mainly at the plasma membrane (Additional file 5: Figure S4b and Figure S4c). Lower expression was achieved for *Olfr166* and *Olfr558* which synthesis was at detection limits of western blot analysis but visible by immunofluorescence. The well-characterized S6 OR was used in all experiments as positive control.

For functional assays we transiently expressed mDA-ORs in heterologous HEK 293 cells in combination with CRE-SEAP reporter plasmid and determined ligand specificity by measuring SEAP quantity in the culture medium in response to odors. Sixteen odor molecules were used for ligand screening including standard odors used for ORs de-orphanization (Additional file 6: Table S2) [17] as well as putative ligands proposed on the basis of sequence similarity between the ORs present in the OlfactionDB (http://molsim.sci.univr.it/OlfactionDB) and queried mDA-ORs plus chemical ligands identified by chemoinformatic approaches (Additional file 7: Figure S5).

**Figure 4:**
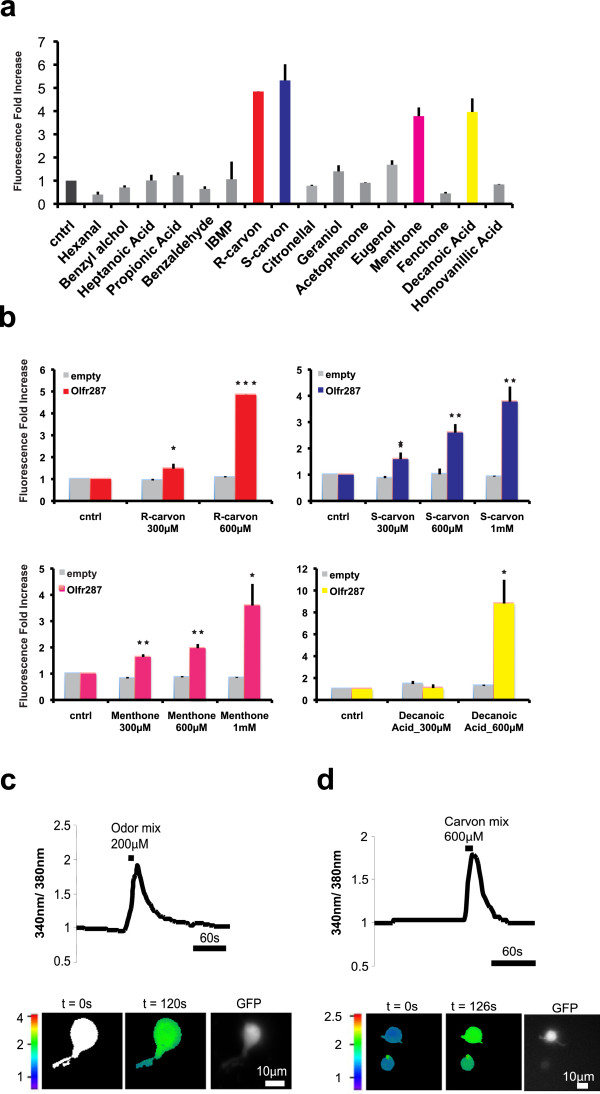
**Selected odors activate mDA-ORs expressed in heterologous cells as well as primary mDA neurons. a)** *Olfr287* was transiently transfected in HEK cells in combination with pCRE-SEAP. After transfection, cells were pulsed with the indicated odor molecules at 600 μM concentration. DMSO was used as negative control (cntrl). mDA-OR activation was measured with fluorimetric assay on culture medium. Odor response was calculated as fold-increase relative to control value. Data indicate mean ± st dev and are calculated on n = 4 independent experiments. **b)** Cells were transfected as in a) and pulsed with increasing concentrations of R-carvone, S-carvone, menthone and decanoic acid. Ringer’s solution or DMSO was used as control (cntrl). mDA-OR activation was calculated as in **a)**. Data indicate mean ± st dev and are calculated on at least three independent experiments. *, p < 0.05; **, p < 0.01; *** p < 0.001. **c)** Ca^2+^ dynamics in primary DA neurons dissociated from the ventral midbrain of TH-GFP mouse. Normalized fluorescence ratio changes (340/380 nm) were measured in DA neurons loaded with Fura-2 AM and challenged with bath-applied odor mixture composed by 16 odorants at 200 μM each (recordings from N ≈ 30 neurons and repeated in n = 2 independent experiments). Images are presented in pseudocolour scale, as indicated. GFP fluorescence of DA neurons is shown. Scale bars indicate 10 μm. **d)** Experiment as in **c)**. Primary neurons were stimulated with (R)- and (S)-carvone mixture at 600 μM each.

*Olfr287* was the only receptor showing a robust, reproducible response to selected odor-like molecules (Figure 4a; Additional file 8: Figure S6). The strongest responses were elicited by S- and R-carvones as well as decanoic acid.

Active odors were then used at increasing concentrations proving their function as agonists at μM levels (Figure 4b). Their specificity was measured in cells transfected with an empty control vector (Figure 4b).

### Isolated primary mDA neurons respond to odor molecules

To assess the physiological response of DA cells to odor-like agonists at mDA-ORs, we used ratiometric Ca^2+^ dynamics evoked by odor stimulation on isolated neurons. Primary mDA neurons were prepared from TH-GFP mice upon enzymatic digestion and mechanical trituration of MB. Isolated neurons were loaded with the Ca^2+^ indicator fura-2 AM and then were challenged with odor mixture (Figure 4c) or a mix of carvone enantiomers (Figure 4d). About one-third of DA neurons used for the analysis showed Ca^2+^ responsiveness upon odor stimulation. In particular, 29.7% were activated by odor mixture (n = 11/37) and 31% (n = 9/29) by carvones. Pulse with odors typically induced a fast onset of the Ca^2+^ signal that lasted 1–2 min (odor mix: cells n = 11, ΔR/R_0_ = 80.5 ± 26; carvone mix: n = 9, ΔR/R_0_ = 73 ± 14.4). Importantly, after odor washout, DA cells showed normal Ca^2+^ transients to high K^+^ solution, indicating that the general excitability and downstream Ca^2+^ entry were intact (data not shown).

### A subset of human ORs is expressed in SN and regulated in PD *post mortem*brains

**Figure 5:**
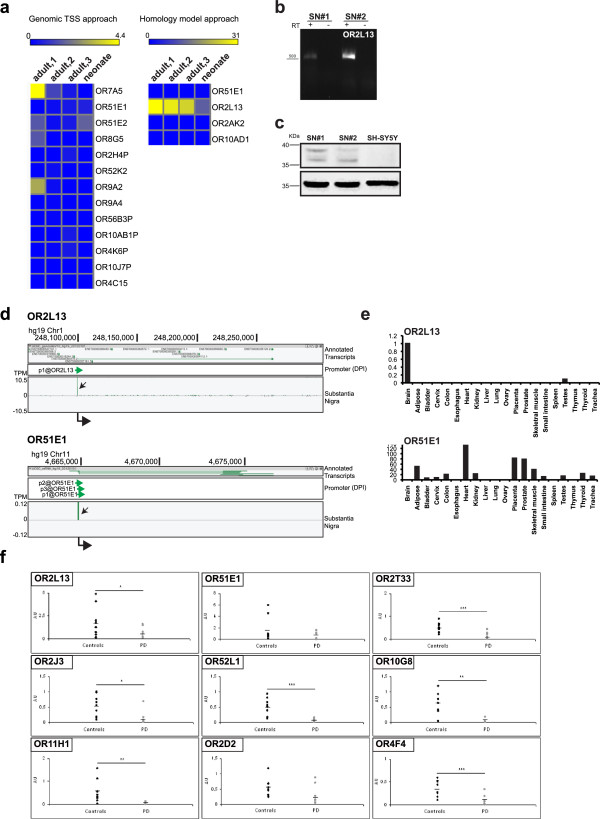
**A subset of human ORs is expressed in SN and down-regulated in PD** ***post mortem*** **brains. a)** FANTOM5 CAGE data in human SN samples. Heat map graphical representation of human ORs expression in adult and neonate samples, as indicated. List of human ORs identified by genomic approach (left) and by homology search (right). **b)** Complete open reading frame for human OR2L13 was cloned from RNA extracted from SN of two individuals (SN#1 and SN#2), not affected by neurodegenerative disorders (controls). **c)** Proteins were extracted from *post mortem* material. Western blot analysis was performed with anti-OR51E1 antibody. Protein extract from SH-SY5Y cells was included as negative control. β-actin was used as loading control. Images are representative of n = 2 independent experiment with n = 2 independent SN samples. **d)** ZENBU genome browser view of CAGE signals for OR2L13 and OR51E1 in human SN. Genomic coordinates are shown on top and scale of expression values (TPM) on the left. Black arrows in each track highlight transcription start sites (TSS). At the bottom, thick arrowheads indicate direction of transcription. **e)** qRT-PCR validation of human mDA-ORs expression in human tissues. A panel of human RNAs from various tissues was used to validate atypical expression of OR2L13 and OR51E1. Brain was arbitrarily set as 1. **f)** qRT-PCR of SN from control and PD *post mortem* brain samples. *p < 0.05; **p < 0.01; ***p < 0.001.

We then aimed to identify ORs in human SN. To this purpose we used two unbiased approaches. First, we took advantage of the large FANTOM5 collection of deep-sequencing datasets [11]. These include four libraries from SN derived from *post mortem* tissues of three human adults and one neonate (Francescatto M, Vitezic M, Rizzu P, Simon-Sanchez J, Andersson R, FANTOM Consortium, Daub CO, Sandelin A, de Hoon MJL, Carninci P, Forrest AR, Heutink P: **A high resolution spatial-temporal promoterome of the human brain**. *Submitted*). To obtain the coordinates of putative promoters for human ORs, we selected windows of 200 bp centered on transcription start sites of mouse OR genes [13] and “lifted” their coordinates over the most recent version of annotated human genome [18]. This analysis pulled out 19 putative coordinates in the human genome for which transcription was evident in any of the SN libraries. We then manually verified each genomic position. 5 of them turned out to be artifacts, as not corresponding to any human OR gene locus (data not shown). The remaining 14 coordinates identified *bona fide* putative human OR TSS (Figure 5a, left panel), with two of them falling in the same OR locus (OR10J7P). Main TSS could be found at the exact positions of annotated RefSeq (OR7A5, OR51E1 and OR51E2), further upstream, between -500 bp and -2000 bp (OR8G5, OR2H4P, OR52K2 and OR9A2), in the intragenic region (OR9A4, OR56B3P, OR10AB1P) or at the 3′-end (OR4K6P). For two of them, the position was far upstream (>5000 bp) of the annotated gene and thus not further investigated (OR10J7P and OR4C15).

As a second approach, we applied sequence similarity comparison with mouse mDA-OR. To this purpose we performed a multiple sequence alignment between human ORs present in the OlfactionDB, for which functional validation exists, and the validated mouse mDA-ORs. Based on this alignment, a maximum-parsimony phylogenetic tree was then built (Additional file 9: Figure S7). We found that highest similarities could be measured between *Olfr558* and human OR51E1 (94% sequence identity), *Olfr166* and OR2L13 (85% identity), *Olfr287* and OR10AD1 (81% identity) and *Olfr316* and OR2AK2 (76% identity).

We then took advantage of FANTOM5 data for human SN to monitor expression of candidate human OR genes using tag counts across the whole locus of interest (sum5end). We thus found evidence of transcription for most of the selected human ORs with TPM values ranging from 31.03 to 0.12 (Figure 5a). Negative counts were present for those ORs for which TSS coordinates were poorly found (OR10J7P and OR4C15) and those with weaker homology (OR10AD1 and OR2AK2). OR2L13 was the top expressed OR. OR51E1, identified by both approaches, could also be found in human SN, although at lower level. We then validated their expression by successfully cloning OR2L13 cDNAs from SN RNA (Figure 5b) and by detecting two bands of the expected molecular weight by using an anti-OR51E1 antibody on human SN extracts (Figure 5c).

Interrogation of the global FANTOM5 human promoter expression atlas (N = 988 libraries) showed that the repertoire of OR identified in the SN has a complex pattern of expression (Additional file 10: Figure S8). Liver is the only organ where none of the analyzed OR is expressed. OR2L13 was strongly enriched in the brain, while OR51E1 was highly expressed in prostate. Their TSS usage is shown in Figure 5d. qRT-PCR analysis of human tissues validated these findings (Figure 5e).

For functional assay, OR2L13 was then transiently expressed in heterologous HEK 293 cells in combination with CRE-SEAP reporter plasmid to monitor SEAP quantity in response of odor stimulation. Membrane localization and size of the ectopically expressed protein were monitored by immunocytochemistry and western blot (Additional file 11: Figure S9a and b). Statistically significant responses were elicited by carvone enantiomers and eugenol although at detection limits (Additional file 11: Figure S9c and d).

Given their expression at the site of neurodegeneration, we monitored OR transcripts in SN from 11 PD and 10 control *post mortem* brains. OR2L13 and OR51E1 expression were analyzed together with a subset of ORs that were previously demonstrated to be down-regulated in the cortex of PD patients [19]. To achieve high sensitivity and specificity, we set up TaqMan assays for each OR gene transcript. First, we could measure the expression of this panel of ORs in the SN of several individuals (controls), further implementing the repertoire of OR transcripts in this brain region (Figure 5f). Then, we found that OR gene expression is down-regulated in the SN of PD patients, as recently reported for the cortex [19], with the only exception of OR51E1, whose decrease is not significant. Down-regulation of human ORs in PD patients cannot be solely due to neuronal cell loss since in the same samples we could detect either unchanged or up-regulated levels of some taste receptors (TASR) [20] that were found expressed in the SN (Additional file 12: Figure S10).

Altogether, our results highlight the expression of a repertoire of OR genes in human SN and their dysregulation in PD.

## Discussion

In the main OE the detection of a vast repertoire of volatile compounds (odors) is accomplished by a large family of ORs on the surface of the cilia of OSNs. Odorants binding to ORs activate G proteins and initiate downstream signaling that leads to Ca^2+^ influx and ultimately to the perception of smell.

Each mature OSN in the OE is thought to express only one allele of a single OR gene—monoallelic and monogenic expression, respectively. A given OR gene is expressed in a mosaic or punctate pattern of OSNs within a characteristic zone of the OE. The transcriptional mechanisms that underlie this extraordinary restriction in gene expression remain unclear. To address this issue, by applying nanoCAGE to mouse OE, we have previously associated TSSs to 955 mouse OR genes, thereby defining a comprehensive picture of their promoter map at a single-base resolution. In contrast with the archetype of the broad shape of >75% of mammalian promoters, OR genes have sharp promoters exhibiting a dominant TSS [13].

A distinctive feature in the topographic organization of the olfactory system is that ORs also plays an instructive role in the axonal convergence of OSN into the olfactory bulb (OB). This function is supported by genetic experiments and by the expression of the OR on the axon termini of the OSN. OR at the growth cone of OSN are capable of binding odors and are coupled to cAMP synthesis and Ca^2+^ influx through cyclic nucleotide gated (CNG) channels. This suggests a potential role of OR activation in axonal convergence and sensory map formation [17, 21].

Interestingly, ORs have been also found to reside in tissues other than those involved in olfaction [22–24]. Several distinct ORs are expressed predominantly or exclusively in spermatogenic cells, where they mediate sperm chemotaxis [25]. Selected ORs accumulate in prostate cancer to inhibit proliferation [26]. *Olfr16*, a well-characterized receptor in mouse, controls chemosensing and motility in sperm [27] as well as regeneration, cell adhesion and migration in muscle [28]. Single ORs were found expressed in layer II pyramidal neurons in the occipital lobe and tightly controlled during development, possibly contributing to axon guidance [29]. More recently, by taking advantage of mRNA-Seq data of 16 human tissues available from Illumina Body Map project 2.0, 111 OR genes were shown expressed outside of OE. Interestingly, OR51E1 and OR2W3 were expressed in all the tissues analyzed while others showed a more restricted pattern with several examples of expression in only one tissue [23]. Therefore, it is becoming evident that non-olfactory cells can hijack ORs as a general signaling pathway to achieve other cell-specific functions.

Here we show that multiple ORs are expressed by mouse mDA neurons, a cell system that is involved in movement control and reward behavior. Interestingly, OR gene expression is also present in other regions as well as outside of the mouse brain. Global transcriptome analysis, cDNA full-length cloning and quantitative RT-PCR indicate that OR transcripts can be also found in human SN.

NanoCAGE [12] is a next-generation sequencing technology for unbiased 5′-end transcriptome profiling and have provided measurement of the expression level of ORs transcripts along with the precise definition of their TSSs from purified mDA cells. Being a single-nucleotide resolution technology, it greatly differs in terms of quantitative and qualitative output from microarray platforms or from PCR screenings based on degenerated oligonucleotides. While the expression of a significant number of *Olfrs* has been confirmed both with nanoCAGE analysis of LCM-purified cells and RT-PCR from MB, some discrepancies have been noticed that may be explained as follows. First, A9 and particularly A10 DA cell groups are highly heterogeneous. On the basis of morphology and connectivity 13 different A10 neuronal cell types have been described. The low associated TPM scores may thus be interpreted as RNAs transcribed by a specific DA subtype leading to expression under detection threshold in total MB analysis. This has been already shown by the comparison of the DeepCAGE data with *in situ* hybridization for the hippocampus [30] and reported for ORs in the gut, where they were expressed exclusively in gastrointestinal enterochromaffin cells, which constitute only a minor proportion of the total intestinal epithelium [31]. Importantly, nanoCAGE data do not offer any description of the anatomy of the transcript. Gisselmann and collegues [23] showed that human ORs give raise to a plethora of transcripts with surprising features: unexpected internal introns, truncated or chimeric RNAs with adjacent genes and 5′ ends derived from distant, previously undescribed TSSs. While we have provided full-length cDNAs of validated mDA-ORs cloned from MB, a detailed analysis of transcript anatomy for every single gene presenting nanoCAGE tags will assess this important issue.

As in OE, validated mDA-ORs present sharp promoters in mDA cells. Most importantly, the very same TSS is often used in both tissues. Sharp promoters without CpG islands are often bound by TFs within a constrained spacing range. ORs promoters are no exceptions showing positional preferences for specific motifs such as for Mef2a, EBF1/HOX and SOX [13]. This transcription factor binding site code can also be relevant for their transcriptional control in mDA cells as well as in the brain. This may suggest that the promoter architecture of ORs may be under evolutionary pressure to drive transcription both in OE and in other tissues strongly challenging the idea that OR expression outside OE is “atypical” or “ectopic”.

Deorphanization of ORs is a very important task that has been attempted at different scales. While virtual High-Throughput Screening (HTS) has been successful to identify novel ligands [32], large efforts have been dedicated to the optimization of heterologous expression systems for efficient ectopic membrane localization of ORs and detection of responses to odor-like molecules. Among the most successful HTSs, 93 odorants have been tested on 464 ORs expressed in heterologous cells identifying agonists for 52 mouse and 10 human ORs [33, 34].

In this study, the screening has been limited to 16 potential ligands and 5 ORs cDNAs. Odor-like molecules have been chosen for representing the major chemical moieties and putative ligands proposed on the basis of sequence similarity between mDA-ORs and those present in the OlfactionDB plus chemical ligands identified by chemoinformatic approaches.

While *Olfr287* was the only ORs showing convincing responses, analysis of *Olfr166* and *Olfr558* were limited by their low expression at the cell membrane.

Despite these limitations we have been able to identify the first odor-like molecule that act on mDA cells. Carvones are monocyclic monoterpene with antioxidant, antimicrobial, anticonvulsant, and antitumor activities. (R)-(-)-carvone is the main constituent of spearmint oil (Mentha spicata var. crispa) while (S)-(+)-carvone is a major component of caraway and dill seed oils. We prove that they elicited a strong response in *Olfr287* expressed in the heterologous system and that they triggered Ca^2+^ responsiveness in solitary mDA neurons. A formal prove that mDA neurons response is mediated by *Olfr287* requires further experiments that are beyond the scope of this work.

These results suggest a potential role of OR activation in mDA physiology. However, the identity of the endogenous ligands for mDA-ORs remains unknown.

A further optimization of heterologous expression of mDA-ORs and a largest repertory of odor-like molecules will probably lead to the discovery of additional agonists at mDA cells and therefore to new molecular tools to manipulate mDA subtypes’ activities.

This concept can be extended to any ORs expressed outside the OE and to any expressing neuronal cell types. Odor-like molecules may thus represent new opportunities for developing neuroactive drugs that act on a defined repertoire of cells. If coupled with radioisotopes, they might also serve as probes for *in vivo* imaging.

Since in the OE the functional activation of ORs requires *Gα*_*olf*_ and *Adcy3*, it is important that we detected the expression of these signaling molecules in mDA neurons as well. Many evidences position *Gα*_*olf*_ at a crucial crossroad of the dopaminergic system in health and disease [35]. So far G*α*_*olf*_--mediated phenotypes have been interpreted for its ability to mediate dopamine receptor 1 signaling in striatal medium spiny neurons [36]. According to our work, it will be also important to assess the contribution of signaling initiated at ORs.

Albeit limited by the small size of patients’ samples available for this study, we have shown that the large majority of human ORs expressed in SN are down-regulated in PD *post mortem* brains. Interestingly, in the same PD samples TASR are up-regulated, thus suggesting that OR down-regulation is not entirely due to neuronal cells loss. For some of the ORs expressed in human SN such as OR51E1, OR2J3 and OR51E2, HTS have led to the identification of potential agonists. It will be thus interesting assaying, respectively, nonanoic acid and butyl butyryllacetate, cys-3-hexen-1-ol and cynnamaldehyde as well as propionic acid for their activities on human DA cells.

Early-stage PD has been associated to a significant smell dysfunction in some monogenic forms as well as in sporadic PD cases, with a prevalence of approximately 90% [37]. Unlike cells in the SN, periglomerular DA cells of the olfactory bulb do not degenerate but increase in number with a concomitant induction of TH expression. The basis for olfactory dysfunction in PD is currently unknown. It remains to be determined whether ORs down-regulation in SN has a role in the olfactory dysfunction observed in PD patients [37].

## Conclusion

By nanoCAGE transcriptome profiling we demonstrate that a subset of ORs is expressed in isolated A9 and A10 mDA neurons. In these cells, odor-like stimuli are able to evoke Ca^2+-^signals. Expression of ORs is also detected in human SN, the site of neurodegeneration in PD, and is found regulated in PD *post mortem* brains.

ORs might thus contribute to the normal physiology of mDA neurons in mammals and potentially be target of pharmacological manipulation with odor-like molecules in disease.

## Methods

### Animals

All animal experiments were performed in accordance with European guidelines for animal care and following SISSA Ethical Committee permissions. Mice were housed and bred in SISSA non-SPF animal facility, with 12 hours dark/light cycles and controlled temperature and humidity. Mice had *ad libitum* access to food and water.

LCM was performed on 12 weeks-old female TH-GFP/21-31 mice [14] (n = 3). C57BL/6 female mice (n = 5), 12 weeks old, were used for *in situ* hybridization and immunohistochemistry experiments. Intra-cardiac perfusions were done under total anaesthesia.

Isolated mDA neurons were prepared from P9-P10 TH-GFP pups (n = 8).

### NanoCAGE library preparation and data analysis

Synthesis of nanoCAGE libraries, sequencing and bioinformatic analysis were carried out as in Plessy *et al*. [12, 13] and Lazarevic *et al*. (Lazarevic D, Bertin N, Franke V, Vlachouli C, Caiazzo M, Plessy C, Akalin A, Vatta P, Simone R, Roncaglia P, Daub CO, Faulkner GJ, Broccoli V, Lenhard B, Carninci P, Gustincich S: **The promotorome of adult dopaminergic neurons of the mouse Substantia Nigra identifies new gene networks for cell conversion**. *Submitted*).

### PCR, quantitative RT-PCR (qRT-PCR) and cloning

Total RNA was extracted from cells and tissue using Trizol (Invitrogen) according to manufacturer’s instruction. RNA was extracted from LCM- purified cells with Absolutely RNA Nanoprep Kit (Stratagene). RNA samples were treated with DNAseI (Ambion). A panel of purified DNAse-treated human tissue-specific RNAs was obtained from Life Sciences (FirstChoice® Human Total RNA Survey Panel). cDNA was prepared from 1 μg of RNA using the iSCRIPT™ cDNA Synthesis Kit (Bio-Rad) according to manufacturer’s instructions. Non-quantitative RT-PCR was performed with standard protocol. qRT-PCR was performed in triplicate using SYBR-Green PCR Master Mix (Applied Biosystem) and an iCycler IQ Real time PCR System (Bio-Rad). Relative gene expression was calculated with ΔΔCt method.

A heat map graphical representation of rescaled normalized fold expression (ΔΔCt/ΔΔCt_MAX_) was obtained using the Matrix2png software (http://www.bioinformatics.ubc.ca/matrix2png/).

Full-length mDA-ORs were cloned from ventral midbrain and inserted into pcDNA3.1-Rho vector kindly provided by Prof. Liberles. Cloned mDA-ORs were verified upon sequencing.

The complete list of oligonucleotides used for PCR and RT-qPCR is in Additional file 13: Table S3. All amplicons were sequenced.

### *In situ*hybridization (ISH)

Sense and antisense probes were generated by *in vitro* transcription from the cDNA encoding *Olfr287*, *Olfr316* and *Olfr558*. Riboprobe synthesis and hybridization were performed as in Carrieri et al. [16]. Probes were labelled with biotin (BIO-labelling mix, Roche) and were used at a concentration of 4 μg/ml at 60°C for 16 h. List of primers used for generating probes is in Additional file 13: Table S3.

### Phylogenetic tree and *in silico*ligand identification

The evolutionary history was inferred using the Maximum Parsimony method. The MP tree was obtained using the Close-Neighbour-Interchange algorithm, with search level 0 in which the initial trees were obtained with the random addition of sequences (10 replicates). The analysis involved 87 amino acid sequences. All positions containing gaps and missing data were eliminated. There were a total of 285 positions in the final dataset. Evolutionary analyses were conducted using MEGA5 [38]. The structures of odorant ligands and their cognate receptors were taken from the OlfactionDB database (http://molsim.sci.univr.it/OlfactionDB). Structurally similar ligands were identified by virtual screening of the ‘ligand.info’ database [39]. This database contains <1,160,000 ligands. Structural similarity was estimated by the Tanimoto’s equation using the ROCS algorithm in the OpenEye suite of programs (http://www.eyesopen.com). For each template, the 100 best hits were selected.

### Cell lines and transfection

Human embryonic kidney 293 cells (HEK 293) were grown as in Carrieri et al. [16]. MN9D-Nurr1^Tet-On^ cells (iMN9D) [40] were kindly provided by Professor Perlmann and maintained as in Biagioli et al. [15].

Transfection was performed with Fugene HD (Promega) following manufacturer’s instructions.

### Western blot

48 h after transfection, cells were lysed in Lysis Buffer (20 mM Tris–HCl pH 7.5, 150 mM NaCl, 1 mM EDTA, 1% TritonX-100). The lysates were incubated 30′ on ice and clarified by centrifugation at 13.000 rpm for 20′. Cell pellets were resuspended in Sample Buffer 2×. Total protein lysates from mouse organs were prepared by homogenization in Lysis Buffer (140 mM NaCl, 5 mM KCl, 5 mM NaHCO_3_, 1.2 mM Na_2_HPO_4_, 1 mM MgCl_2_, 20 mM HEPES pH 7.4, 10 mM dextrose, 1.8 mM CaCl_2_). Proteins from human samples were extracted using Trizol reagent and following manufacturer’s instructions. Equal amount of proteins were separated in 10% SDS-polyacrilamide gel and transferred to nitrocellulose membrane. Immunoblotting was performed with the following primary antibodies: anti-Rhodopsin tag (Novus Biological), anti-βactin (A5441, Sigma) and anti-OR51E1 (Thermo Scientific). Signals were revealed after incubation with secondary antibodies conjugated with horseradish peroxidase by using Immobilon (Millipore).

### Immunohistochemistry and immunocytochemistry

Immunohistochemistry was performed as previously described on 16 μm-thick cryo-slices prepared from 12 weeks old C57BL/6 mice (n = 3) [15]. Primary antibodies anti-OR51E1 1:1000 (Novus Biologicals) and anti-TH 1:1000 (SIGMA) were used.

For immunocytochemistry experiments, 48 h after transfection, cells were fixed in 4% paraformaldehyde for 10 minutes, washed with PBS and treated with 0.1 M glycine for 4 minutes. After washing, cells were blocked in the non-permeabilizing buffer (0.2% BSA, 1% NGS, in PBS). Cells were incubated with anti-Rho tag antibody 1:1000 in blocking solution for 90 minutes at room temperature. After washes in PBS, cells were incubated with secondary antibody Alexa Fluor-488 (Molecular Probes-Invitrogen,) for 1 h. Nuclei were visualized with 1 μg/ml DAPI. Cells were mounted with Vectashield (Vector laboratories) and analyzed at confocal microscope (Leica).

### mDA-ORs functional assays

Functional assays were performed as described previously [41]. Briefly, 80,000 HEK 293 cells were plated in 24-well plates and co-transfected with 400 ng of pCDNA3.1-Rho mDA-OR construct and 400 ng of pCRE-SEAP reporter plasmid. 16 hours after transfection cells were treated with odors for 48 hours. SEAP was detected using GreatEscape SEAP kit (Clontech) according to manufacturer’s instructions. Fluorescence was measured with a Fluorimeter Spectramax M5 multi-mode microplate reader. For test compounds, see Additional file 6: Table S2.

### Calcium Imaging in primary mDA neurons

Isolation of mDA neurons was performed as described previously [15, 42]. Briefly, ventral midbrain was isolated and the pieces containing the SN were enzymatically dissociated with Papain (SIGMA) under continuous oxygenation (5% CO2 and 95% O2 gas mixture) with slow stirring at 35°. After 40 minutes, the reaction was stopped with 1 mg/ml trypsin inhibitor (Sigma) and the pieces were triturated by using p1000 tip and glass pasteur pipette. The cell suspensions were centrifuged at 1000 rpm for 3 min and the pellet was resuspended. Dissociated neurons were plated on the slides coated with poly-lysine (1 mg/ml, SIGMA). After 3 hours, Ca^2+^ imaging was performed.

Dissociated neurons were incubated with 8 μM fura-2 AM, 80 μg/ml Pluronic F127 and sulphinil pyrazone 250 μM (Molecular Probes) for 20 minutes.

Neurons were constantly perfused in Ringer’s solution (3 ml/min) except during stimulation applied for 4-10s. Stimuli were a mixture of odors (citronella, citralva, (+)-carvone, (-) carvone, menthone, geraniol, eugenol, acetophenone, benzyl alcohol, benzaldeheyde, propionic acid, heptanoic acid, IBMP) or a mixture containing both R- and S-carvone enantiomers. All odorants are from Sigma Aldrich.

Changes in intracellular Ca^2+^ were visualized using 380 nm and 340 nm excitation filters and 510/40 nm emission filter and were acquired every 3 s. using a Cell^R^ system. Changes in fluorescence (340 nm /380 nm) were expressed as R/R_0_ where R is the ratio at time t and R_0_ is the ratio at time = 0 s. Responses (%) were evaluated as ΔR/R_0_ × 100 where ΔR = R-R_0_.

### Bioinformatic analysis of olfactory receptors in FANTOM5 collection of human libraries

We selected windows of 200 bp on the mouse genome, centered on transcription start sites of olfactory receptors detected using nanoCAGE [13] and “lifted” their coordinates over the human genome version 19 [18] to obtain putative promoters for human olfactory receptors. For three pairs of receptors, *Olfr55/Olfr239*, *Olfr216/Olfr317*, and *Olfr1507/Olfr1508*, the lift produced the same coordinates, and we discarded each second member of the pairs. The expression levels of these putative human OR promoters was calculated by counting the number of CAGE tags from the FANTOM 5 promoter expression atlas [11] starting in these windows, using scripts available upon request, based on tabix [43] and bedtools [44]. Out of 499 regions, 100 had more tags than the 3rd quartile and 15 had more than 100 tags, an arbitrary cutoff that we chose after visual inspection of the data in FANTOM5’s instance of the ZENBU genomic browser [45], which showed that lower scores were enriched for apparently spurious accumulation of tags near pseudogenes.

### Human samples

Brain tissue was obtained from the Institute of Neuropathology HUB-ICO-IDIBELL (University Hospital of Bellvitge- IDIBELL Foundation) Bio-Bank following the guidelines of Spanish legislation on this matter and in compliance with the Helsinki Declaration (http://www.wma.net/en/30publications/10policies/b3/index.html). Samples were dissected at autopsy with the informed consent of patients or their relatives and the institutional approval of the local Ethics Committee (HUB-ICO/CEIC), signed by Dr. Enric Sospedra Martinez.

Cases analyzed included 10 controls and 11 PD cases [19]. Samples of SN were dissected at the time of autopsy, and immediately frozen and stored at -80°C until use. The purification of RNA was carried out with RNeasy Lipid Tissue Mini Kit (Qiagen, DE) following the protocol provided by the manufacturer. During purification, samples were treated with RNase-free DNase Set (Qiagen, DE) to avoid later amplification of genomic DNA. The concentration of each sample was obtained from A260 measurements with Nanodrop 1000. RNA integrity was tested using the Agilent 2100 BioAnalyzer (Agilent, US). The retrotranscriptase reaction was carried out using a High Capacity cDNA Archive kit (Applied Biosystems, US) following the protocol provided by the supplier. Parallel reactions for each RNA sample were run in the absence of MultiScribe Reverse Transcriptase to assess the degree of contaminating genomic DNA.

TaqMan PCR assays for each gene were performed in duplicate on cDNA samples in 384-well optical plates using an ABI Prism 7900 Sequence Detection System (Applied Biosystems, US). For each 20 μl TaqMan reaction, 9 μl cDNA was mixed with 1 μl 20× TaqMan Gene Expression Assays and 10 μl of 2× TaqMan Universal polymerase chain reaction (PCR) Master Mix (Applied Biosystems, US). The reactions were carried out using the following parameters: 50°C for 2 min, 95°C for 10 min, and 40 cycles of 95°C for 15 s and 60°C for 1 min. Finally, all TaqMan PCR data were captured using the Sequence Detector Software (SDS version 1.9, Applied Biosystems, US). Probes used in this study are listed as follows:

OR2J3: ACCGCCAAGTAGATCACTTTTTCTG

OR52L1: CTCAGCAGATCCGCCAGCGAGTGCT

OR51E1: TACGGTTGAGCCTCTACCTGCCTGG

OR2L13: CTCCAAGCCCAGTTACAGCAGAAAG

OR2T33: AACGGTGGCTGGGGACGTGTGTAAA

OR11H1: CACTGGGAGACATAAGGCCTTCTCT

OR2D2: GTGAGGCCCCTGCACTATTGATCTT

OR4F4: TATACACACTGAGGAACAAAGACAT

TAS2R4: CACCATTTACTGTGGCCTTCATCTC

TAS2R5: TTTCTTGTTTCCTCTGGGATGCTGA

TAS2R10: ACCACAGCCATCTATCCCTGGGGTC

TAS2R13: CACCATTTACTGTGGCCTTCATCTC

TAS2R14: TTTGTCCCTGGCAATGTTTCTTCTC

TAS2R50: AGTCCTAGGAGGCTGCGGAATGACC

Samples were analyzed with the double delta CT (ΔΔCT) method. Delta CT (ΔCT) values represent normalized target gene levels with respect to the internal controls (GUSB, Glucuronidase beta: GCTACTACTTGAAGATGGTGATCGC; XPNPEP1, X-prolyl aminopeptidase 1: CAAAGAGTGCGACTGGCTCAACAAT; and AARS, alanyl-tRNA synthetase: GCAAAATTTGGGGCTGGATGACACC). Reference genes were selected because they are very efficient in replicating microarray target gene expression in human *post mortem* brain tissue. ΔΔCT values were calculated as the ΔCT of each test sample minus the mean ΔCT of the calibrator samples for each target gene. The fold change was calculated using the equation 2^(-ΔΔCT)^. Results were subjected to statistical analysis as described below.

### Statistical analysis

Statistical analyses were performed with paired two-tailed Student’s t-test. Results are mean (*n* ≥3) ± standard deviation (s.d.). Number of replicas in each experiment is further described in figure legends. qRT-PCR data on human samples were analyzed by two-way ANOVA followed by Student’s *t*-test when required. Differences between mean values were considered statistically significant * p < 0.05; ** p < 0.01; *** p < 0.001.

### Data access

NanoCAGE sequences have been submitted to the DNA DataBank of Japan Sequence Read Archive (DRA) under accession number DRA000475. Data will be available upon release of an accompanying manuscript (Lazarevic D, Bertin N, Franke V, Vlachouli C, Caiazzo M, Plessy C, Akalin A, Vatta P, Simone R, Roncaglia P, Daub CO, Faulkner GJ, Broccoli V, Lenhard B, Carninci P, Gustincich S: **The promotorome of adult dopaminergic neurons of the mouse Substantia Nigra identifies new gene networks for cell conversion**. *Submitted*). This work is part of the FANTOM5 project. Data downloads and genomic tools are summarized here http://fantom.gsc.riken.jp/5/.

## Authors’ information

the FANTOM Consortium: Email: fantom5-secretariat@gsc.riken.jp.

## Electronic supplementary material

Additional file 1: Table S1: List of mDA-ORs. Complete list of ORs identified by nanoCAGE in A9 and A10 neurons. Expression values (TPM) measured for each receptor is indicated in the appropriate column. No expression is also shown (-). Results from non-quantitative PCR validation in RNA extracted from total midbrain (MB) or from laser capture microdissected (LCM) neurons are indicated (-, negative; +, positive; NT, not tested). (PDF 125 KB)

Additional file 2: Figure S1: Expression of OR-mediated signal transduction elements in mouse mDA neurons. **a)** nanoCAGE datasets. UCSD Genome browser view of *Gα*_*olf*_ and *Adcy3* expression in A9, A10. Tracks from nanoCAGE of olfactory epithelium (from Plessy et. al, Genome Research, [13]) are included for comparison (OE). Initiation of annotated RefSeq is shown. Black arrows indicate transcription start sites (TSS). Genomic coordinates are shown on top and expression values (TPM) on the left. Direction of transcription is indicated by a thick arrowhead at the bottom of each panel. **b)** FANTOM5 mouse datasets. Zenbu genome browser view of *Gα*_*olf*_ and *Adcy3* expression in neurons from SN and in olfactory brain. TPM values are shown on the left. (PDF 298 KB)

Additional file 3: Figure S2: Specificity of expression of ORs in mDA neurons. Specificity of expression of *Olfr287*, *Olfr316* and *Olfr558* transcripts (green) by ISH in A9 and A10 DA neurons is verified with control sense probes. DA neurons in SN are visualized by anti-TH immuno-staining (red). Nuclei are shown in blue (DAPI). Scale bars indicate 20 μm. Data are representative of n = 3 independent experiments. (PDF 674 KB)

Additional file 4: Figure S3: Endogenous OR protein is expressed in mDA neurons and in mouse brain. **a)** Anti-OR51E1 antibody recognized *Olfr558* (green) expressed in heterologous HEK 293 cells. Immunofluorescence in non-permeabilizing conditions. Nuclei are visualized with DAPI (blue). Scale bars indicate 38 μm. White arrows highlight transfected cells. **b)** Endogenous OR protein is detected in A9 and A10 mDA neurons and in the cortex. mDA neurons were visualized with anti-TH (red) and OR with anti-OR51E1 specific antibody (green). Nuclei are stained with DAPI (blue). Scale bars indicate 20 μm. Images are representative of n = 3 independent experiments. (PDF 517 KB)

Additional file 5: Figure S4: Overexpression of mDA-ORs in iMN9D and HEK 293 cells. mDA-ORs were cloned from the ventral midbrain into pCDN3.1-Rho tag vector. Plasmids were transiently transfected in iMN9D cells. Expression of mDA-ORs (*olfr166*, *olfr287*, *olfr316*, *olfr558* and *olfr1344*) was verified by western blotting (n = 5) **(a)** and immunofluorescence (n = 3) **(b)** with anti-Rho antibody. pCDN3.1- empty vector and *S6* OR expressing plasmid were used as negative and positive controls, respectively. **c)** HEK 293 cells were transiently transfected with the indicated mDA-ORs and tested as in **b**. (PDF 559 KB)

Additional file 6: Table S2: List of odors. Complete list of odors used in this study. Odor formulation and concentration of stock solution are indicated. (PDF 135 KB)

Additional file 7: Figure S5: Evolutionary and chemoinformatics characterization of mDA-ORs. **a)** Model of phylogenetic tree distribution of mDA-ORs. **b)** Putative ligands for each receptor based on ligands of the closest homologue in the phylogenetic tree and by a chemoinformatic search. (PDF 752 KB)

Additional file 8: Figure S6: Analysis of mDA-ORs responses to selected odors. mDA-ORs were transiently transfected in HEK cells in combination with pCRE-SEAP. After transfection, cells were pulsed with odor molecules at 600 μM concentration. Nonanoic acid was used at 1 mM. Ringer’s solution or DMSO was used as control. mDA-OR activation was measured with fluorimetric assay on culture medium. mDA-ORs used in this assays (*olfr166*, *olfr316*, *olfr558* and *olfr1344*) are indicated. Data indicate mean ± st dev and are calculated on four independent experiments. (PDF 160 KB)

Additional file 9: Figure S7: Sequence homology strategy for human mDA-ORs. Model of phylogenetic tree distribution of mouse mDA-ORs for the identification of human homologues. (PDF 131 KB)

Additional file 10: Figure S8: Expression of human mDA-ORs in selected human FANTOM5 hCAGE libraries. Color-coded representation of OR expression in human FANTOM5 hCAGE libraries. Values are expressed in tag per million (TPM). SN libraries are in red; tissue libraries that were validated by qRT-PCR are in blue. (PDF 158 KB)

Additional file 11: Figure S9: Analysis of OR2L13 response to selected odors. Expression of human OR2L13 in HEK cells was verified by immunofluorescence **(a)** and western blotting **(b)** with anti-Rho antibody. pCDN3.1-empty vector and *S6* OR expressing plasmid were used as negative and positive controls, respectively. For functional assays, OR2L13 was transiently transfected in HEK cells in combination with pCRE-SEAP. After transfection, cells were challenged with odor molecules at 600 μM concentration **(c)** or at the indicated quantities **(d)**. Ringer’s solution or DMSO was used as control. mDA-OR activation was measured with fluorometric assay on culture medium. Data indicate mean ± st dev and are calculated on two independent experiments. (PDF 396 KB)

Additional file 12: Figure S10: A subset of human Taste Receptors is regulated in PD. qRT-PCR of SN from control and PD *post mortem* brain samples. Data indicate mean ± stdev. *p < 0.05. (PDF 117 KB)

Additional file 13: Table S3: List of primers. Complete list of oligonucleotides used in this study for non-quantitative PCR, quantitative RT-PCR, cloning and *in situ* hybridization. (PDF 54 KB)

## Abbreviations

CAGE: Cap analysis of gene expression
TPM: Tag per million
LCM: Laser capture microdissection
TH: Tyrosine hydroxylase
mDA neurons: Mesencephalic dopaminergic neurons
PD: Parkinson’s disease
OR: Olfactory receptor
OE: Olfactory epithelium
MB: Ventral midbrain
OSN: Olfactory sensory neurons
SN: Substantia Nigra
VTA: Ventral tegmental area
SEAP: Secreted alkaline phosphatase
CRE: cAMP response element
TASR: Taste receptors
HTS: High throughput screening.

## References

[1] Bjorklund A, Dunnett SB (2007). Fifty years of dopamine research. Trends Neurosci.

[2] Bjorklund A, Dunnett SB (2007). Dopamine neuron systems in the brain: an update. Trends Neurosci.

[3] Hirsch E, Graybiel AM, Agid YA (1988). Melanized dopaminergic neurons are differentially susceptible to degeneration in Parkinson’s disease. Nature.

[4] Meyer-Lindenberg A, Miletich RS, Kohn PD, Esposito G, Carson RE, Quarantelli M, Weinberger DR, Berman KF (2002). Reduced prefrontal activity predicts exaggerated striatal dopaminergic function in schizophrenia. Nat Neurosci.

[5] Chung CY, Seo H, Sonntag KC, Brooks A, Lin L, Isacson O (2005). Cell type-specific gene expression of midbrain dopaminergic neurons reveals molecules involved in their vulnerability and protection. Hum Mol Genet.

[6] Greene JG, Dingledine R, Greenamyre JT (2005). Gene expression profiling of rat midbrain dopamine neurons: implications for selective vulnerability in parkinsonism. Neurobiol Dis.

[7] Grimm J, Mueller A, Hefti F, Rosenthal A (2004). Molecular basis for catecholaminergic neuron diversity. Proc Natl Acad Sci U S A.

[8] Shiraki T, Kondo S, Katayama S, Waki K, Kasukawa T, Kawaji H, Kodzius R, Watahiki A, Nakamura M, Arakawa T, Fukuda S, Sasaki D, Podhajska A, Harbers M, Kawai J, Carninci P, Hayashizaki Y (2003). Cap analysis gene expression for high-throughput analysis of transcriptional starting point and identification of promoter usage. Proc Natl Acad Sci U S A.

[9] Carninci P, Sandelin A, Lenhard B, Katayama S, Shimokawa K, Ponjavic J, Semple CA, Taylor MS, Engstrom PG, Frith MC, Forrest AR, Alkema WB, Tan SL, Plessy C, Kodzius R, Ravasi T, Kasukawa T, Fukuda S, Kanamori-Katayama M, Kitazume Y, Kawaji H, Kai C, Nakamura M, Konno H, Nakano K, Mottagui-Tabar S, Arner P, Chesi A, Gustincich S, Persichetti F (2006). Genome-wide analysis of mammalian promoter architecture and evolution. Nat Genet.

[10] Gustincich S, Batalov S, Beisel KW, Bono H, Carninci P, Fletcher CF, Grimmond S, Hirokawa N, Jarvis ED, Jegla T, Kawasawa Y, LeMieux J, Miki H, Raviola E, Teasdale RD, Tominaga N, Yagi K, Zimmer A, Hayashizaki Y, Okazaki Y (2003). Analysis of the mouse transcriptome for genes involved in the function of the nervous system. Genome Res.

[11] Forrest AR, Kawaji H, Rehli M, Baillie JK, de Hoon MJ, Lassmann T, Itoh M, Summers KM, Suzuki H, Daub CO, Kawai J, Heutink P, Hide W, Freeman TC, Lenhard B, Bajic VB, Taylor MS, Makeev VJ, Sandelin A, Hume DA, Carninci P, Hayashizaki Y, FANTOM Consortium and the RIKEN PMI and CLST (DGT) (2014). A promoter-level mammalian expression atlas. Nature.

[12] Plessy C, Bertin N, Takahashi H, Simone R, Salimullah M, Lassmann T, Vitezic M, Severin J, Olivarius S, Lazarevic D, Hornig N, Orlando V, Bell I, Gao H, Dumais J, Kapranov P, Wang H, Davis CA, Gingeras TR, Kawai J, Daub CO, Hayashizaki Y, Gustincich S, Carninci P (2010). Linking promoters to functional transcripts in small samples with nanoCAGE and CAGEscan. Nat Methods.

[13] Plessy C, Pascarella G, Bertin N, Akalin A, Carrieri C, Vassalli A, Lazarevic D, Severin J, Vlachouli C, Simone R, Faulkner GJ, Kawai J, Daub CO, Zucchelli S, Hayashizaki Y, Mombaerts P, Lenhard B, Gustincich S, Carninci P (2012). Promoter architecture of mouse olfactory receptor genes. Genome Res.

[14] Sawamoto K, Nakao N, Kobayashi K, Matsushita N, Takahashi H, Kakishita K, Yamamoto A, Yoshizaki T, Terashima T, Murakami F, Itakura T, Okano H (2001). Visualization, direct isolation, and transplantation of midbrain dopaminergic neurons. Proc Natl Acad Sci U S A.

[15] Biagioli M, Pinto M, Cesselli D, Zaninello M, Lazarevic D, Roncaglia P, Simone R, Vlachouli C, Plessy C, Bertin N, Beltrami A, Kobayashi K, Gallo V, Santoro C, Ferrer I, Rivella S, Beltrami CA, Carninci P, Raviola E, Gustincich S (2009). Unexpected expression of alpha- and beta-globin in mesencephalic dopaminergic neurons and glial cells. Proc Natl Acad Sci U S A.

[16] Carrieri C, Cimatti L, Biagioli M, Beugnet A, Zucchelli S, Fedele S, Pesce E, Ferrer I, Collavin L, Santoro C, Forrest AR, Carninci P, Biffo S, Stupka E, Gustincich S (2012). Long non-coding antisense RNA controls Uchl1 translation through an embedded SINEB2 repeat. Nature.

[17] Maritan M, Monaco G, Zamparo I, Zaccolo M, Pozzan T, Lodovichi C (2009). Odorant receptors at the growth cone are coupled to localized cAMP and Ca2+ increases. Proc Natl Acad Sci U S A.

[18] Hinrichs AS, Karolchik D, Baertsch R, Barber GP, Bejerano G, Clawson H, Diekhans M, Furey TS, Harte RA, Hsu F, Hillman-Jackson J, Kuhn RM, Pedersen JS, Pohl A, Raney BJ, Rosenbloom KR, Siepel A, Smith KE, Sugnet CW, Sultan-Qurraie A, Thomas DJ, Trumbower H, Weber RJ, Weirauch M, Zweig AS, Haussler D, Kent WJ (2006). The UCSC genome browser database: update 2006. Nucleic Acids Res.

[19] Garcia-Esparcia P, Schluter A, Carmona M, Moreno J, Ansoleaga B, Torrejon-Escribano B, Gustincich S, Pujol A, Ferrer I (2013). Functional genomics reveals dysregulation of cortical olfactory receptors in Parkinson disease: novel putative chemoreceptors in the human brain. J Neuropathol Exp Neurol.

[20] Ansoleaga B, Garcia-Esparcia P, Llorens F, Moreno J, Aso E, Ferrer I (2013). Dysregulation of brain olfactory and taste receptors in AD, PSP and CJD, and AD-related model. Neuroscience.

[21] Lodovichi C, Belluscio L (2012). Odorant receptors in the formation of the olfactory bulb circuitry. Physiology (Bethesda).

[22] Feldmesser E, Olender T, Khen M, Yanai I, Ophir R, Lancet D (2006). Widespread ectopic expression of olfactory receptor genes. BMC Genomics.

[23] Flegel C, Manteniotis S, Osthold S, Hatt H, Gisselmann G (2013). Expression profile of ectopic olfactory receptors determined by deep sequencing. PLoS ONE.

[24] Zhang X, Rogers M, Tian H, Zhang X, Zou DJ, Liu J, Ma M, Shepherd GM, Firestein SJ (2004). High-throughput microarray detection of olfactory receptor gene expression in the mouse. Proc Natl Acad Sci U S A.

[25] Spehr M, Gisselmann G, Poplawski A, Riffell JA, Wetzel CH, Zimmer RK, Hatt H (2003). Identification of a testicular odorant receptor mediating human sperm chemotaxis. Science.

[26] Neuhaus EM, Zhang W, Gelis L, Deng Y, Noldus J, Hatt H (2009). Activation of an olfactory receptor inhibits proliferation of prostate cancer cells. J Biol Chem.

[27] Fukuda N, Yomogida K, Okabe M, Touhara K (2004). Functional characterization of a mouse testicular olfactory receptor and its role in chemosensing and in regulation of sperm motility. J Cell Sci.

[28] Griffin CA, Kafadar KA, Pavlath GK (2009). MOR23 promotes muscle regeneration and regulates cell adhesion and migration. Dev Cell.

[29] Otaki JM, Yamamoto H, Firestein S (2004). Odorant receptor expression in the mouse cerebral cortex. J Neurobiol.

[30] Valen E, Pascarella G, Chalk A, Maeda N, Kojima M, Kawazu C, Murata M, Nishiyori H, Lazarevic D, Motti D, Marstrand TT, Tang MH, Zhao X, Krogh A, Winther O, Arakawa T, Kawai J, Wells C, Daub C, Harbers M, Hayashizaki Y, Gustincich S, Sandelin A, Carninci P (2009). Genome-wide detection and analysis of hippocampus core promoters using DeepCAGE. Genome Res.

[31] Braun T, Voland P, Kunz L, Prinz C, Gratzl M (2007). Enterochromaffin cells of the human gut: sensors for spices and odorants. Gastroenterology.

[32] Triballeau N, Van Name E, Laslier G, Cai D, Paillard G, Sorensen PW, Hoffmann R, Bertrand HO, Ngai J, Acher FC (2008). High-potency olfactory receptor agonists discovered by virtual high-throughput screening: molecular probes for receptor structure and olfactory function. Neuron.

[33] Mainland JD, Keller A, Li YR, Zhou T, Trimmer C, Snyder LL, Moberly AH, Adipietro KA, Liu WL, Zhuang H, Zhan S, Lee SS, Lin A, Matsunami H (2014). The missense of smell: functional variability in the human odorant receptor repertoire. Nat Neurosci.

[34] Saito H, Chi Q, Zhuang H, Matsunami H, Mainland JD (2009). Odor coding by a Mammalian receptor repertoire. Sci Signal.

[35] Nishi A, Kuroiwa M, Shuto T (2011). Mechanisms for the modulation of dopamine d(1) receptor signaling in striatal neurons. Front Neuroanat.

[36] Zhuang X, Belluscio L, Hen R (2000). G(olf)alpha mediates dopamine D1 receptor signaling. J Neurosci.

[37] Doty RL (2012). Olfactory dysfunction in Parkinson disease. Nat Rev Neurol.

[38] Tamura K, Peterson D, Peterson N, Stecher G, Nei M, Kumar S (2011). MEGA5: molecular evolutionary genetics analysis using maximum likelihood, evolutionary distance, and maximum parsimony methods. Mol Biol Evol.

[39] von Grotthuss M, Koczyk G, Pas J, Wyrwicz LS, Rychlewski L (2004). Ligand. Info small-molecule Meta-Database. Comb Chem High Throughput Screen.

[40] Hermanson E, Joseph B, Castro D, Lindqvist E, Aarnisalo P, Wallen A, Benoit G, Hengerer B, Olson L, Perlmann T (2003). Nurr1 regulates dopamine synthesis and storage in MN9D dopamine cells. Exp Cell Res.

[41] Liberles SD, Buck LB (2006). A second class of chemosensory receptors in the olfactory epithelium. Nature.

[42] Puopolo M, Raviola E, Bean BP (2007). Roles of subthreshold calcium current and sodium current in spontaneous firing of mouse midbrain dopamine neurons. J Neurosci.

[43] Li H (2011). Tabix: fast retrieval of sequence features from generic TAB-delimited files. Bioinformatics.

[44] Quinlan AR, Hall IM (2010). BEDTools: a flexible suite of utilities for comparing genomic features. Bioinformatics.

[45] Severin J, Lizio M, Harshbarger J, Kawaji H, Daub CO, Hayashizaki Y, Bertin N, Forrest AR (2014). Interactive visualization and analysis of large-scale sequencing datasets using ZENBU. Nat Biotechnol.

