# Supplementary material for: Mesencephalic dopaminergic neurons express a repertoire of olfactory receptors and respond to odorant-like molecules
Source: BMC Genomics. 2014 Aug 27;15(1):729. doi: 10.1186/1471-2164-15-729 (PMC4161876; doi:10.1186/1471-2164-15-729)
Supplement: Supplementary file 1 — Additional file 1: Table S1: List of mDA-ORs. Complete list of ORs identified by nanoCAGE in A9 and A10 neurons. Expression values (TPM) measured for each receptor is indicated in the appropriate column. No expression is also shown (-). Results from non-quantitative PCR validation in RNA extracted from total midbrain (MB) or from laser capture microdissected (LCM) neurons are indicated (-, negative; +, positive; NT, not tested). (PDF 125 KB) [file 12864_2013_6425_MOESM1_ESM.pdf]

	nanoCAGE libraries		PCR validation	
**receptor_ID**	**A9**	**A10**	**MB**	**LCM neurons**
**Olfr78**	0.26	-	-	NT
**Olfr118**	1.8	-	-	NT
**Olfr130**	1.33	-	-	NT
**Olfr234**	-	0.13	-	NT
**Olfr287**	-	3.49	+	+
**Olfr299**	-	3.3	+	-
**Olfr316**	4.29	-	+	+
**Olfr420**	-	4.41	-	NT
**Olfr449**	1.31	-	-	NT
**Olfr502**	-	5.72	-	NT
**Olfr723**	2.71	6.98	-	NT
**Olfr883**	-	1.74	+	+
**Olfr885**	0.15	1	-	NT
**Olfr909**	-	4.19	-	NT
**Olfr958**	-	3.88	-	NT
**Olfr1129**	-	1.96	-	NT
**Olfr1288**	-	1.3	+	-
**Olfr1344**	multiple tags	multiple tags	+	+
**Olfr1443**	-	0.34	-	NT

Supplementary Table S1. List of mDA-ORs. Complete list of ORs identified by nanoCAGE in A9 and A10 neurons. Expression values (TPM) measured for each receptor is indicated in the appropriate column. No expression is also shown (-). Results from non-quantitative PCR validation in RNA extracted from total midbrain (MB) or from laser capture microdissected (LCM) neurons are indicated (-, negative; +, positive; NT, not tested).
