# Supplementary material for: Mesencephalic dopaminergic neurons express a repertoire of olfactory receptors and respond to odorant-like molecules
Source: BMC Genomics. 2014 Aug 27;15(1):729. doi: 10.1186/1471-2164-15-729 (PMC4161876; doi:10.1186/1471-2164-15-729)
Supplement: Supplementary file 2 — Additional file 2: Figure S1: Expression of OR-mediated signal transduction elements in mouse mDA neurons. a) nanoCAGE datasets. UCSD Genome browser view of Gα olf and Adcy3 expression in A9, A10. Tracks from nanoCAGE of olfactory epithelium (from Plessy et. al, Genome Research, [13]) are included for comparison (OE). Initiation of annotated RefSeq is shown. Black arrows indicate transcription start sites (TSS). Genomic coordinates are shown on top and expression values (TPM) on the left. Direction of transcription is indicated by a thick arrowhead at the bottom of each panel. b) FANTOM5 mouse datasets. Zenbu genome browser view of Gα olf and Adcy3 expression in neurons from SN and in olfactory brain. TPM values are shown on the left. (PDF 298 KB) [file 12864_2013_6425_MOESM2_ESM.pdf]

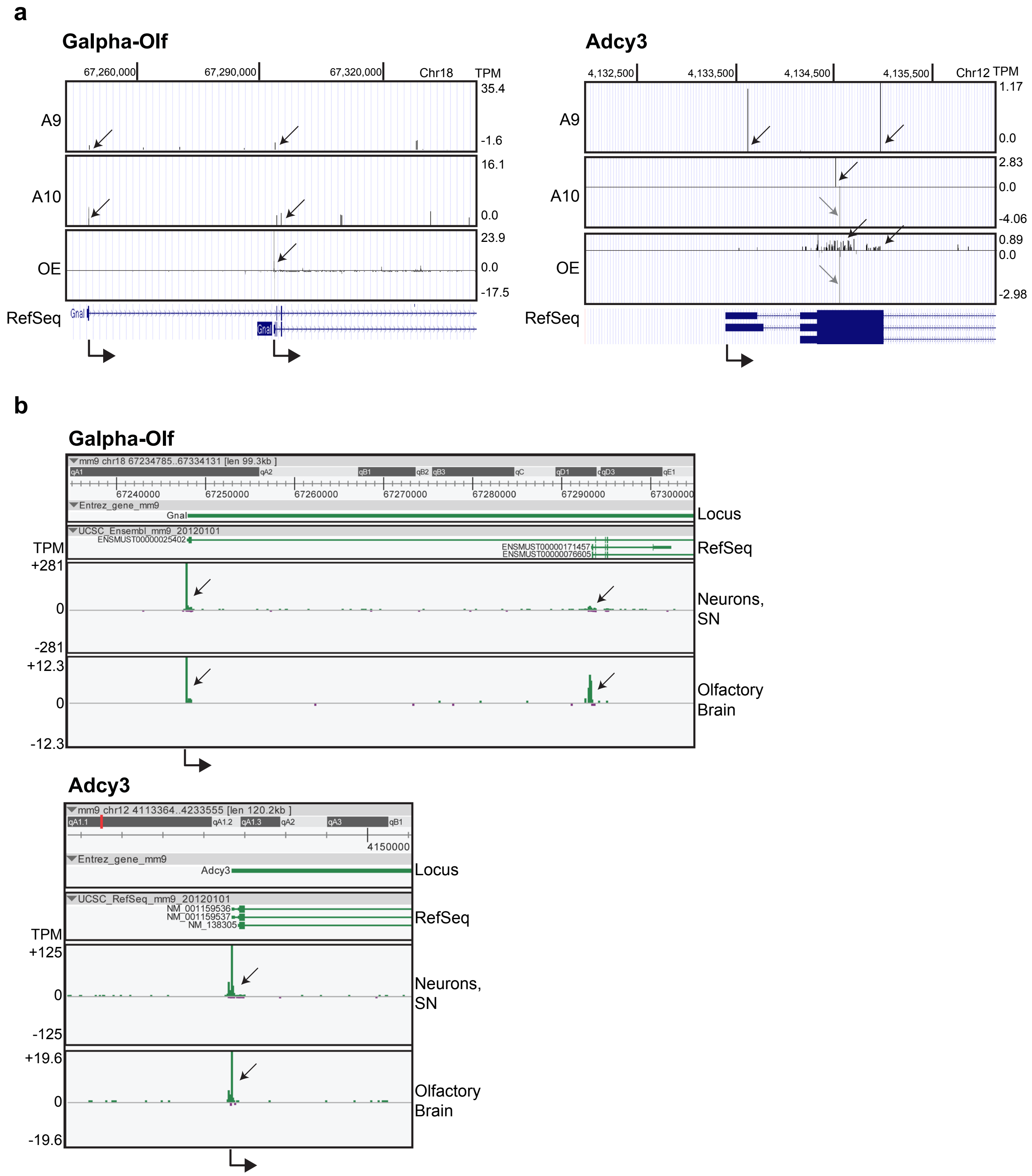

Supplementary Figure S1. Expression of OR-mediated signal transduction elements in mouse mDA neurons. **a)** nanoCAGE datasets. UCSD Genome browser view of *Gαolf* and *Adcy3* expression in A9, A10. Tracks from nanoCAGE of olfactory epithelium (from Plessy et. al., Genome Research, 2012) are included for comparison (OE). Initiation of annotated RefSeq is shown. Black arrows indicate transcription start sites (TSS). Genomic coordinates are shown on top and expression values (TPM) on the left. Direction of transcription is indicated by a thick arrowhead at the bottom of each panel. **b)** FANTOM5 mouse datasets. Zenbu genome browser view of *Gαolf* and *Adcy3* expression in neurons from SN and in olfactory brain. TPM values are shown on the left.
