# Supplementary material for: Mesencephalic dopaminergic neurons express a repertoire of olfactory receptors and respond to odorant-like molecules
Source: BMC Genomics. 2014 Aug 27;15(1):729. doi: 10.1186/1471-2164-15-729 (PMC4161876; doi:10.1186/1471-2164-15-729)
Supplement: Supplementary file 3 — Additional file 3: Figure S2: Specificity of expression of ORs in mDA neurons. Specificity of expression of Olfr287, Olfr316 and Olfr558 transcripts (green) by ISH in A9 and A10 DA neurons is verified with control sense probes. DA neurons in SN are visualized by anti-TH immuno-staining (red). Nuclei are shown in blue (DAPI). Scale bars indicate 20 μm. Data are representative of n = 3 independent experiments. (PDF 674 KB) [file 12864_2013_6425_MOESM3_ESM.pdf]

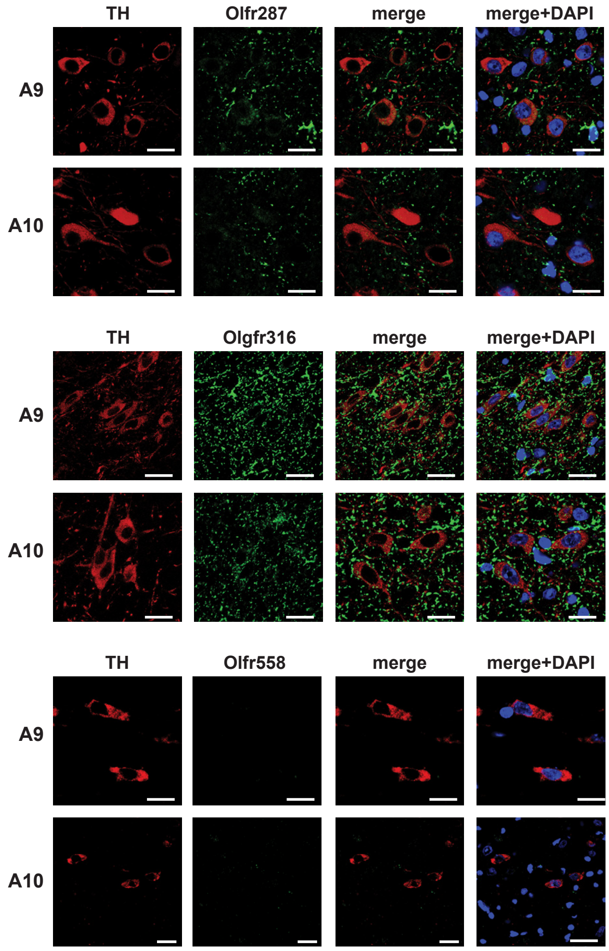

Supplementary Figure S2. Specificity of expression of ORs in mDA neurons. Specificity of expression of *Olfr287*, *Olfr316* and *Olfr558* transcripts (green) by ISH in A9 and A10 DA neurons is verified with control sense probes. DA neurons in SN are visualized by anti-TH immuno-staining (red). Nuclei are shown in blue (DAPI). Scale bars indicate 20 µm. Data are representative of n=3 independent experiments.
