# Supplementary material for: Mesencephalic dopaminergic neurons express a repertoire of olfactory receptors and respond to odorant-like molecules
Source: BMC Genomics. 2014 Aug 27;15(1):729. doi: 10.1186/1471-2164-15-729 (PMC4161876; doi:10.1186/1471-2164-15-729)
Supplement: Supplementary file 4 — Additional file 4: Figure S3: Endogenous OR protein is expressed in mDA neurons and in mouse brain. a) Anti-OR51E1 antibody recognized Olfr558 (green) expressed in heterologous HEK 293 cells. Immunofluorescence in non-permeabilizing conditions. Nuclei are visualized with DAPI (blue). Scale bars indicate 38 μm. White arrows highlight transfected cells. b) Endogenous OR protein is detected in A9 and A10 mDA neurons and in the cortex. mDA neurons were visualized with anti-TH (red) and OR with anti-OR51E1 specific antibody (green). Nuclei are stained with DAPI (blue). Scale bars indicate 20 μm. Images are representative of n = 3 independent experiments. (PDF 517 KB) [file 12864_2013_6425_MOESM4_ESM.pdf]

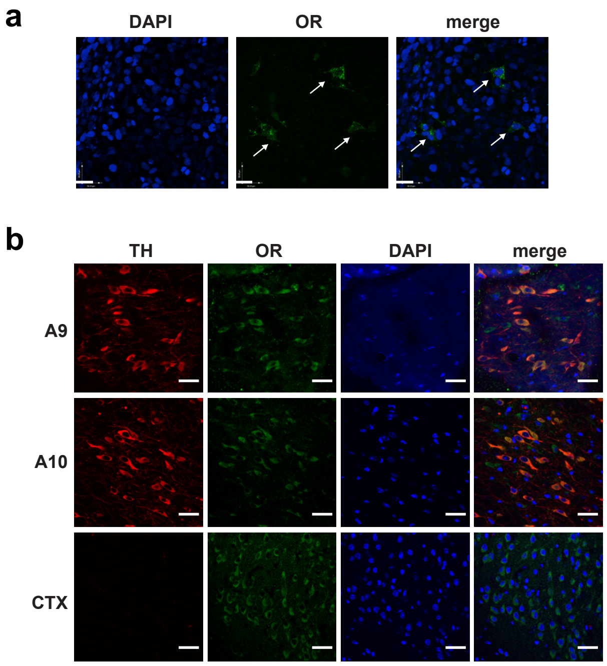

Supplementary Figure S3. Endogenous OR protein is expressed in mDA neurons and in mouse brain. a) Anti-OR51E1 antibody recognized *Olfr558* (green) expressed in heterologous HEK 293 cells. Immunofluorescence in non-permeabilizing conditions. Nuclei are visualized with DAPI (blue). Scale bars indicate 38 µm. White arrows highlight transfected cells. **b)** Endogenous OR protein is detected in A9 and A10 mDA neurons and in the cortex. mDA neurons were visualized with anti-TH (red) and OR with anti-OR51E1 specific antibody (green). Nuclei are stained with DAPI (blue). Scale bars indicate 20 µm. Images are representative of n=3 independent experiments.
