# Supplementary material for: Mesencephalic dopaminergic neurons express a repertoire of olfactory receptors and respond to odorant-like molecules
Source: BMC Genomics. 2014 Aug 27;15(1):729. doi: 10.1186/1471-2164-15-729 (PMC4161876; doi:10.1186/1471-2164-15-729)
Supplement: Supplementary file 5 — Additional file 5: Figure S4: Overexpression of mDA-ORs in iMN9D and HEK 293 cells. mDA-ORs were cloned from the ventral midbrain into pCDN3.1-Rho tag vector. Plasmids were transiently transfected in iMN9D cells. Expression of mDA-ORs (olfr166, olfr287, olfr316, olfr558 and olfr1344) was verified by western blotting (n = 5) (a) and immunofluorescence (n = 3) (b) with anti-Rho antibody. pCDN3.1- empty vector and S6 OR expressing plasmid were used as negative and positive controls, respectively. c) HEK 293 cells were transiently transfected with the indicated mDA-ORs and tested as in b. (PDF 559 KB) [file 12864_2013_6425_MOESM5_ESM.pdf]

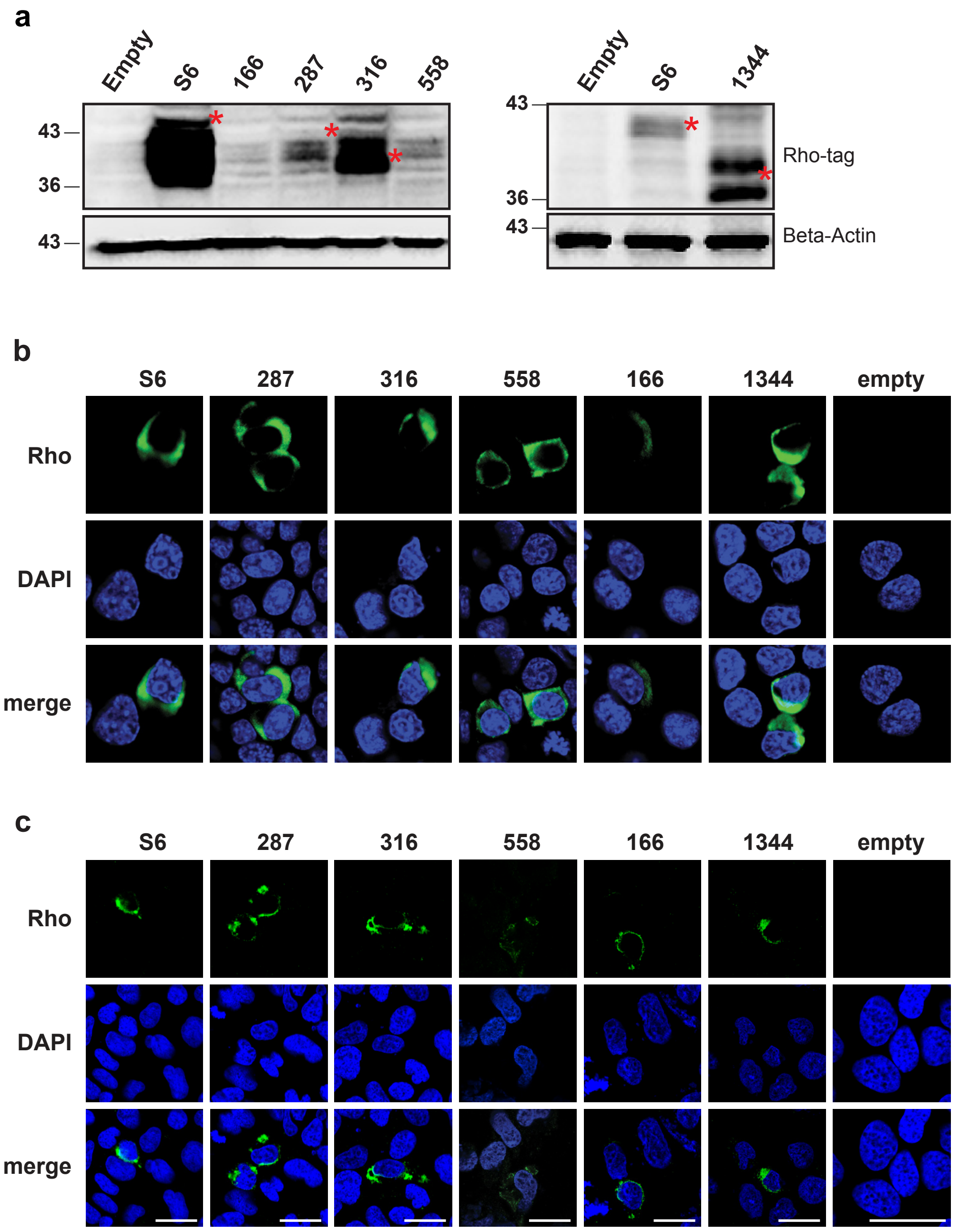


**Supplementary Figure S4. Overexpression of mDA-ORs in iMN9D cells.** mDA-ORs were cloned from the ventral midbrain into pCDN3.1-Rho tag vector. Plasmids were transiently transfected in iMN9D cells. Expression of mDA-ORs (*olfr166*, *olfr287*, *olfr316*, *olfr558* and *olfr1344*) was verified by western blotting (n=5) **(a)** and immunofluorescence (n=3) **(b)** with anti-Rho antibody. pCDN3.1- empty vector and *S6* OR expressing plasmid were used as negative and positive controls, respectively. **c)** HEK 293 cells were transiently transfected with the indicated mDA-ORs and tested as in b.
