# Supplementary material for: Mesencephalic dopaminergic neurons express a repertoire of olfactory receptors and respond to odorant-like molecules
Source: BMC Genomics. 2014 Aug 27;15(1):729. doi: 10.1186/1471-2164-15-729 (PMC4161876; doi:10.1186/1471-2164-15-729)
Supplement: Supplementary file 6 — Additional file 6: Table S2: List of odors. Complete list of odors used in this study. Odor formulation and concentration of stock solution are indicated. (PDF 135 KB) [file 12864_2013_6425_MOESM6_ESM.pdf]

Olous odor	Stock concentration (M)
Hexanal	8.15M
Benzyl Alcohol	9.6M
Heptanoic acid	6.9M
Propionic acid	13M
Benzaldehyde	9.8M
IBMP	5.8M
R-carvone	6.25M
S-carvone	6.13M
Citronellal	5.3M
Geraniol	5.58M
Acetophenone	8.3M
Eugenol	6.1M
Menthone	5.6M
Fenchone	6.17M
**Powdered odors**	**Stock concentration**
Decanoic acid	172.26 g/mol
Homovanillic acid	182 g/mol

Supplementary Table S2. List of odors. Complete list of odors used in this study. Odor formulation and concentration of stock solution are indicated.
