# Supplementary material for: Mesencephalic dopaminergic neurons express a repertoire of olfactory receptors and respond to odorant-like molecules
Source: BMC Genomics. 2014 Aug 27;15(1):729. doi: 10.1186/1471-2164-15-729 (PMC4161876; doi:10.1186/1471-2164-15-729)
Supplement: Supplementary file 7 — Additional file 7: Figure S5: Evolutionary and chemoinformatics characterization of mDA-ORs. a) Model of phylogenetic tree distribution of mDA-ORs. b) Putative ligands for each receptor based on ligands of the closest homologue in the phylogenetic tree and by a chemoinformatic search. (PDF 752 KB) [file 12864_2013_6425_MOESM7_ESM.pdf]

a

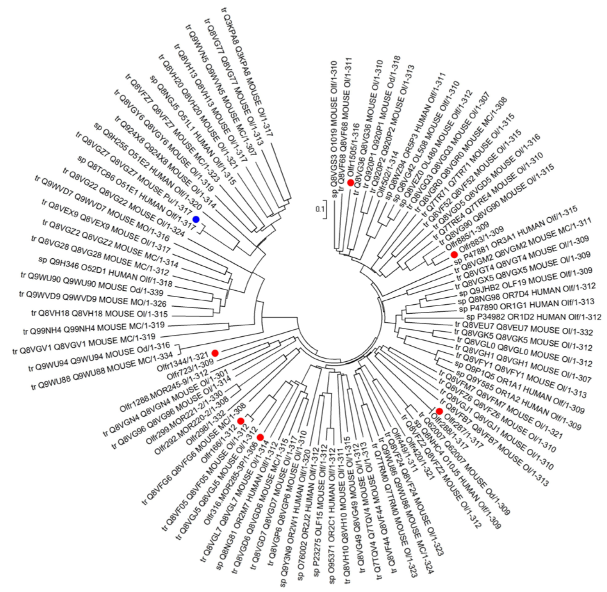

b

RECEPTOR		% SEQ. IDENTITY	CLOSEST HOMOLOGOUS			PUTATIVE LIGAND
MOR18-1	Olfr558	100	Q8VGZ7	MOR18-1	Olfr558	Nonanoic acid
MOR270-1	Olfr166	95	Q8VF05	MOR271-1	Olfr168	(+)-Fenchone
MOR162-6	Olfr883	79	Q8VG90	MOR162-1	Olfr983	4-Chromanone
MOR285-3P	Olrf316	54	Q8VF05	MOR271-1	Olfr168	(+)-Fenchone
		52	Q920P2	MOR174-9	Olfr73	(+)-Fenchone
MOR211-4P	Olf1505	51	Q8VGS3	MOR180-1	Olfr1019	Prenyl acetate
		49	Q8VF68	MOR205-1	Olfr221	Dihydrojasmone
MOR124-1	Olrfr1344	46	Q8VG49	MOR260-1	Olfr715	Decanoic acid
MOR286-1	Olfr287	45	Q8VF44	MOR259-1	Olfr1519	2-Coumaranone

Supplementary Figure S5. Evolutionary and chemoinformatics characterization of mDA-ORs. a) Model of phylogenetic tree distribution of mDA-ORs. **b)** Putative ligands for each receptor based on ligands of the closest homologue in the phylogenetic tree and by a chemoinformatic search.
