# Supplementary material for: Mesencephalic dopaminergic neurons express a repertoire of olfactory receptors and respond to odorant-like molecules
Source: BMC Genomics. 2014 Aug 27;15(1):729. doi: 10.1186/1471-2164-15-729 (PMC4161876; doi:10.1186/1471-2164-15-729)
Supplement: Supplementary file 8 — Additional file 8: Figure S6: Analysis of mDA-ORs responses to selected odors. mDA-ORs were transiently transfected in HEK cells in combination with pCRE-SEAP. After transfection, cells were pulsed with odor molecules at 600 μM concentration. Nonanoic acid was used at 1 mM. Ringer’s solution or DMSO was used as control. mDA-OR activation was measured with fluorimetric assay on culture medium. mDA-ORs used in this assays (olfr166, olfr316, olfr558 and olfr1344) are indicated. Data indicate mean ± st dev and are calculated on four independent experiments. (PDF 160 KB) [file 12864_2013_6425_MOESM8_ESM.pdf]

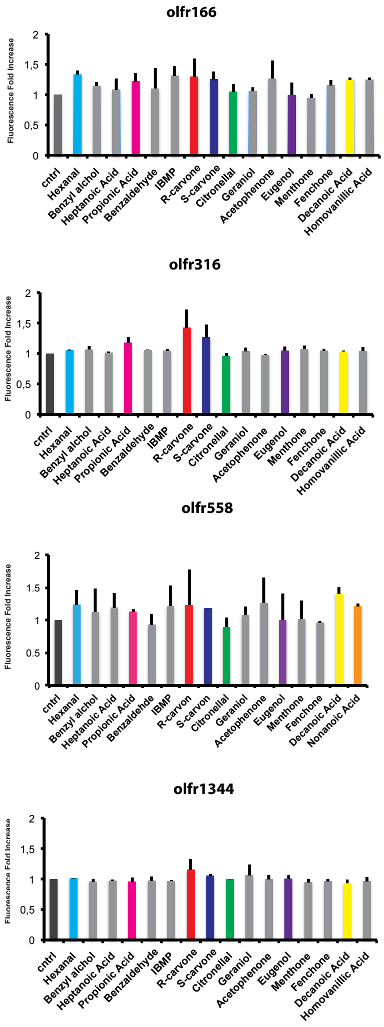


**Supplementary Figure S6. Analysis of mDA-ORs responses to selected odors.** mDA-ORs were transiently transfected in HEK cells in combination with pCRE-SEAP. After transfection, cells were pulsed with odor molecules at 600 μM concentration. Nonanoic acid was used at 1mM. Ringer's solution or DMSO was used as control. mDA-OR activation was measured with fluorimetric assay on culture medium. mDA-ORs used in this assays (*olfr166*, *olfr316*, *olfr558* and *olfr1344*) are indicated. Data indicate mean ± st dev and are calculated on four independent experiments.
