# Supplementary material for: Mesencephalic dopaminergic neurons express a repertoire of olfactory receptors and respond to odorant-like molecules
Source: BMC Genomics. 2014 Aug 27;15(1):729. doi: 10.1186/1471-2164-15-729 (PMC4161876; doi:10.1186/1471-2164-15-729)
Supplement: Supplementary file 9 — Additional file 9: Figure S7: Sequence homology strategy for human mDA-ORs. Model of phylogenetic tree distribution of mouse mDA-ORs for the identification of human homologues. (PDF 131 KB) [file 12864_2013_6425_MOESM9_ESM.pdf]

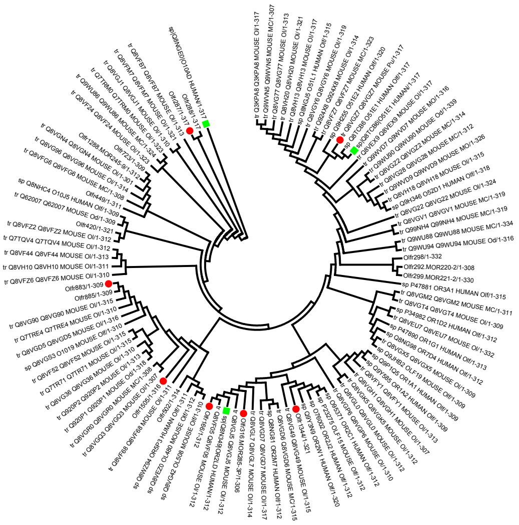

Supplementary Figure S7. Sequence homology strategy for human mDA-ORs. Model of phylogenetic tree distribution of mouse mDA-ORs for the identification of human homologues.
