# Supplementary material for: Mesencephalic dopaminergic neurons express a repertoire of olfactory receptors and respond to odorant-like molecules
Source: BMC Genomics. 2014 Aug 27;15(1):729. doi: 10.1186/1471-2164-15-729 (PMC4161876; doi:10.1186/1471-2164-15-729)
Supplement: Supplementary file 10 — Additional file 10: Figure S8: Expression of human mDA-ORs in selected human FANTOM5 hCAGE libraries. Color-coded representation of OR expression in human FANTOM5 hCAGE libraries. Values are expressed in tag per million (TPM). SN libraries are in red; tissue libraries that were validated by qRT-PCR are in blue. (PDF 158 KB) [file 12864_2013_6425_MOESM10_ESM.pdf]

Brain tissues and primary cells

Experiment	OR7A5	OR51E1	OR51E2	OR8G5	OR2HP4	OR52K2	OR9A2	OR9A4	OR56B3P	OR10AB1P	OR4K6P	OR10J7P	OR4C15	OR2L13	OR10AD1	OR2AK2
amygdala - adult, donor10196 : CNhs13793 ctss [tpm]	0	0.81	2.42	0	0	0	0	0	0	0	0	0	0	20.93	0	0
amygdala, adult, donor10252 : CNhs12311 ctss [tpm]	0	0.22	0.22	0.11	0	0	0	0	0.11	0	0	0	0	27.24	0	0.11
brain, adult, donor1 : CNhs11796 ctss [tpm]	0	0.8	0.8	0	0	0	0	0	0	0	0	0	0	27.95	0	0
brain, adult, pool1 : CNhs10617 ctss [tpm]	0	0.09	0.53	0	0	0	0	0	0	0	0	0	0	22.86	0	0
brain, fetal, pool1 : CNhs11797 ctss [tpm]	0	0	1.84	0	0	0	0	0	0	0	0	0	0	5.52	0	0
caudate nucleus - adult, donor10196 : CNhs13802 ctss [tpm]	0	0	0.72	0	0	0	0	0	0	0	0	0	0	18.1	0	0
caudate nucleus, adult, donor10252 : CNhs12321 ctss [tpm]	0	0.2	0	0	0	0	0.1	0	0	0	0	0	0	19.06	0	0.1
caudate nucleus, adult, donor10258 : CNhs14232 ctss [tpm]	0	0	0	0	0	0	0.48	0	0	0.48	0	0	0	23.96	0	0
cerebellum - adult, donor10196 : CNhs13799 ctss [tpm]	0	0	1.29	0	0	0	0	0	0	0	0	0	0	18.09	0	0
cerebellum, adult, donor10252 : CNhs12323 ctss [tpm]	0.17	0.17	0.25	0.08	0	0	0	0	0	0	0.08	0	0	9.22	0	0
cerebellum, adult, pool1 : CNhs11795 ctss [tpm]	0	0.25	0	0	0	0	0	0.25	0	0	0	0	0	16.66	0.25	0
cerebral meninges, adult : CNhs12840 ctss [tpm]	0.21	0.64	1.07	0	0	0	0	0	0	0	0	0	0	16.06	0	0
cerebrospinal fluid, donor2 : CNhs13437 ctss [tpm]	0	0.71	0.36	0.71	0	0	0	0	0	0.36	0.71	0.36	0	13.88	0	0.36
corpus callosum, adult, pool1 : CNhs10649 ctss [tpm]	0	0.27	0.2	0	0	0	0.27	0	0	0	0	0	0	15.9	0	0.14
diencephalon, adult : CNhs12610 ctss [tpm]	0.46	0.23	0.46	0	0	0.23	0.23	0	0	0	0	0	0	27.63	0	0
frontal lobe, adult, pool1 : CNhs10647 ctss [tpm]	0	0.32	0.32	0	0	0	0	0	0	0	0.06	0	0	20.68	0.06	0.06
globus pallidus - adult, donor10196 : CNhs13801 ctss [tpm]	0	0.99	0	0	0	0	0.99	0	0	0	0	0	0	17.88	0	0.5
globus pallidus, adult, donor10252 : CNhs12319 ctss [tpm]	0.55	0.44	0.11	0.11	0	0.11	0.22	0.11	0	0	0	0	0.11	26.64	0.11	0
globus pallidus, adult, donor10258 : CNhs14549 ctss [tpm]	0.37	5.02	5.51	0.98	0.24	0	0.61	0.73	0.73	0.24	0.73	0	0.37	43.71	0.24	0.49
hippocampus - adult, donor10196 : CNhs13795 ctss [tpm]	0	0	0	0	0	0	0	0	0	0	0	0	0	24.16	0	1.21
hippocampus, adult, donor10252 : CNhs12312 ctss [tpm]	0.19	0.1	0.1	0	0.1	0.1	0	0	0	0	0	0	0	23.72	0	0.19
hippocampus, adult, donor10258 : CNhs14227 ctss [tpm]	0	0.39	0	0	0	0	0.39	0	0	0	0	0	0	31.27	0	0.39
hippocampus, newborn, donor10223 : CNhs14081 ctss [tpm]	0	0.3	0.6	0	0	0	0.3	0	0	0	0.9	0.3	0	16.77	0	0
insula, adult, pool1 : CNhs10646 ctss [tpm]	0	0.31	0.56	0	0	0	0.12	0	0	0	0	0	0	23.29	0	0.37
locus coeruleus - adult, donor10196 : CNhs13808 ctss [tpm]	0.49	0.29	0.2	0	0.1	0	0.2	0	0	0.1	0	0	0	39.51	0.1	0.2
locus coeruleus, adult, donor10252 : CNhs12322 ctss [tpm]	0.22	0.32	0.86	0	0.65	0	0.11	0.11	0	0	0	0	0	30.54	0	0.11
locus coeruleus, adult, donor10258 : CNhs14550 ctss [tpm]	0	0.37	1.12	0	0.19	0	0	0	0	0	0	0	0	51.86	0.19	0.74
medial frontal gyrus - adult, donor10196 : CNhs13796 ctss [tpm]	0	0.71	0	0	0	0	0	0	0	0	0	0	0	36.4	0	0
medial frontal gyrus, adult, donor10252 : CNhs12310 ctss [tpm]	0.29	0.29	0.67	0.19	0	0	0.29	0	0	0.1	0	0	0	39.71	0	0.48
medial temporal gyrus - adult, donor10196 : CNhs13809 ctss [tpm]	0.09	0	0.26	0	0	0	0	0	0.09	0	0	0	0	46.07	0	0.43
medial temporal gyrus, adult, donor10252 : CNhs12316 ctss [tpm]	0.47	0.38	0.57	0	0	0.19	0.19	0	0	0.19	0.09	0	0	40.7	0	0.38
medulla oblongata - adult, donor10196 : CNhs13800 ctss [tpm]	0.42	0.84	0.84	0.42	0.42	0	0	0	0	0	0	0	0	20.65	0	0.42
medulla oblongata, adult, donor10252 : CNhs12315 ctss [tpm]	0.29	0.77	0.48	0.1	0.19	0	0.19	0	0	0	0	0	0.1	16.21	0	0
medulla oblongata, adult, pool1 : CNhs10645 ctss [tpm]	0	0.72	1.11	0	0	0	0.07	0	0	0	0	0	0	21.12	0	0
Neural stem cells, donor1 : CNhs11063 ctss [tpm]	0.42	3.18	4.01	0.55	0	0	0.14	0	0.42	0.14	0.28	0.28	0.42	21.03	0.28	0
Neural stem cells, donor2 : CNhs11384 ctss [tpm]	0.27	4.01	5.87	1.34	0	0	0.53	0.27	0	0.27	0.53	0.53	1.07	29.37	0	0
Neurons, donor1 : CNhs12338 ctss [tpm]	0.5	4.79	8.32	1.01	0.25	0	0.5	0	0.25	0.76	0.25	0.5	0.76	23.45	0.76	0.5
Neurons, donor2 : CNhs12726 ctss [tpm]	0	0.51	1.88	0.51	0	0	0.17	0	0.17	0.17	0.17	0.17	0.68	15.24	0.17	0.17
Neurons, donor3 : CNhs13815 ctss [tpm]	0.64	4.66	4.66	1.91	0	0.21	0.21	0	0	1.06	0.42	0.21	1.06	30.09	0	0
nucleus accumbens, adult, pool1 : CNhs10644 ctss [tpm]	0.14	0.27	0.47	0	0	0	0.07	0	0	0	0	0	0	22.58	0	0.14
occipital cortex - adult, donor10196 : CNhs13798 ctss [tpm]	0	0	0	0	0	0	0	0	0	0	0	0	0	42.5	0	1.18
occipital cortex, adult, donor10252 : CNhs12320 ctss [tpm]	0.1	0.2	1.27	0.29	0	0	0	0	0	0.1	0.1	0	0.1	59.47	0	0.68
occipital lobe, adult, donor1 : CNhs11787 ctss [tpm]	0.49	0.16	1.46	0	0	0	0	0	0	0.16	0	0	0	44.13	0	0.16
occipital lobe, fetal, donor1 : CNhs11784 ctss [tpm]	0	0	0	0	0	0	0	0	0	0	0	0	0	8.6	0	0
occipital pole, adult, pool1 : CNhs10643 ctss [tpm]	0.07	0.52	0.45	0	0	0	0	0	0	0	0	0	0	21.53	0	0
olfactory region, adult : CNhs12611 ctss [tpm]	0	0.13	0.26	0	0	0	0	0	0	0.13	0.13	0	0	44.89	0	0.26
Oligodendrocyte - precursors, donor1 : CNhs12586 ctss [tpm]	0	1.26	1.26	0.63	0	0	0	0	0	0	0	0	0	5.65	0	0
optic nerve, donor1 : CNhs13449 ctss [tpm]	0.7	1.75	3.86	1.4	0	0	0.7	0.7	0.35	0	0.35	0.7	0.35	24.9	0.35	0
paracentral gyrus, adult, pool1 : CNhs10642 ctss [tpm]	0	0.14	0.92	0	0	0.14	0.07	0	0	0	0	0	0	26.73	0	0.14
parietal cortex, adult, donor10258 : CNhs14226 ctss [tpm]	0	0.55	0	0	0	0	0	0	0	0	0	0	0	21.61	0	0
parietal lobe - adult, donor10196 : CNhs13797 ctss [tpm]	0	0	0	0	0	0	0	0	0	0.63	0	0	0	32.79	0	0
parietal lobe, adult, donor10252 : CNhs12317 ctss [tpm]	0.14	0.29	1.29	0	0	0	0	0	0	0.14	0.29	0	0	42.96	0	0.57
parietal lobe, adult, pool1 : CNhs10641 ctss [tpm]	0.14	0.27	1.02	0	0	0	0.07	0	0	0	0	0	0	33.1	0	0.27
parietal lobe, fetal, donor1 : CNhs11782 ctss [tpm]	0	0	0	0	0	0	0	0	0	0	0	0	0	10.34	0.32	0
pons, adult, pool1 : CNhs10640 ctss [tpm]	0.07	0.34	0.07	0	0	0	0	0	0	0.14	0	0	0	33.46	0	0.34
postcentral gyrus, adult, pool1 : CNhs10638 ctss [tpm]	0	0.07	0.97	0	0	0	0.07	0	0	0.07	0	0	0	26.47	0	0.28
putamen, adult, donor10196 : CNhs12324 ctss [tpm]	0.13	0.39	0.78	0	0	0	0.13	0.13	0	0.13	0	0	0.13	23.39	0	0.13
putamen, adult, donor10252 : CNhs13912 ctss [tpm]	0	0	0	0	0	0	0.8	0	0	0	0	0	0	17.66	0	0
putamen, adult, donor10258, tech_rep1 : CNhs14225 ctss [tpm]	0	0.89	0.89	2.66	0	0	0	0	0	0	0	0	0	17.75	0	0.89
putamen, adult, donor10258, tech_rep2 : CNhs14618 ctss [tpm]	0.18	0.18	0.18	0	0	0	0	0	0	0	0	0	0	28.59	0	0
retina, adult, pool1 : CNhs10636 ctss [tpm]	0.16	1.97	27.6	0	0	0	0.08	0.08	0	0	0	0	0	6.06	0	0
Schwann Cells, donor1 : CNhs12073 ctss [tpm]	0	0	0	0	0	0	0	0	0	0	0	0	0	0.52	0	0
Schwann Cells, donor2 : CNhs12345 ctss [tpm]	4.31	0	4.31	0	0	0	0	0	0	0	0	0	0	8.61	0	0
Schwann Cells, donor3 : CNhs12621 ctss [tpm]	0	0	0	0	0	0	0	0	0	0	0	0.62	0	0.31	0	0
spinal cord - adult, donor10196 : CNhs13807 ctss [tpm]	0.14	0.56	0.14	0	0	0	0.28	0	0	0.14	0.28	0.14	0	29.96	0	0
spinal cord, adult, donor10252 : CNhs12227 ctss [tpm]	0.49	0.73	1.22	0	0.24	0.49	0.49	0	0	0	0	0	0	18.32	0	0
spinal cord, adult, donor10258 : CNhs14222 ctss [tpm]	0	0	0.44	0	0	0	0	0	0	0.44	0	0	0	24.01	0	0.89
spinal cord, fetal, donor1 : CNhs11764 ctss [tpm]	0	0.35	1.41	0	0	0	0	0	0	0	0	0	0	11.29	0	0
substantia nigra - adult, donor10196 : CNhs13803 ctss [tpm]	4.43	0	1.48	1.48	0	0	2.96	0	0	0	0	0	0	31.03	0	0
substantia nigra, adult, donor10252 : CNhs12318 ctss [tpm]	1.22	0.24	0.24	0	0	0.24	0.24	0.12	0.12	0	0.12	0	0	28.46	0	0.24
substantia nigra, adult, donor10258 : CNhs14224 ctss [tpm]	0.42	0.42	0.42	0	0.42	0	0	0	0	0.42	0.42	0	0	25.6	0	0
substantia nigra, newborn, donor10223 : CNhs14076 ctss [tpm]	0	0.57	1.7	0	0	0	0	0	0	0	0	0	0	11.35	0	0
temporal lobe, adult, pool1 : CNhs10637 ctss [tpm]	0	0.21	0.48	0	0	0	0	0	0	0	0	0	0	12.87	0	0.07
temporal lobe, fetal, donor1, tech_rep1 : CNhs11772 ctss [tpm]	0	0.32	0	0	0	0	0	0	0	0	0	0	0	15.51	0	0
temporal lobe, fetal, donor1, tech_rep2 : CNhs12996 ctss [tpm]	0	0	0	0	0	0	0.25	0	0	0	0	0	0	13.57	0	0
thalamus - adult, donor10196 : CNhs13794 ctss [tpm]	0.63	0	0	0	0	0	0	0.63	0	0.63	0	0	0	26.64	0	0.63
thalamus, adult, donor10252 : CNhs12314 ctss [tpm]	0.34	0.26	0.17	0.09	0.17	0	0	0	0	0	0	0.09	0	24.23	0	0.09
thalamus, adult, donor10258, tech_rep1 : CNhs14223 ctss [tpm]	0	0	0	0	0	0	0	0	0	0	0	0	0	23.94	0	0.96
thalamus, adult, donor10258, tech_rep2 : CNhs14551 ctss [tpm]	0.11	0.89	0.56	0.22	0.45	0	0.67	0.11	0	0	0	0	0.11	37.17	0	0.22

Non-brain Tissues

Experiment	OR7A5	OR51E1	OR51E2	OR8G5	OR2HP4	OR52K2	OR9A2	OR9A4	OR56B3P	OR10AB1P	OR4K6P	OR10J7P	OR4C15	OR2L13	OR10AD1	OR2AK2
achilles tendon, donor2 : CNhs13435 ctss [tpm]	0.34	2.37	5.08	1.69	0	0	0	0.34	0.34	0.68	1.69	0.34	0.34	37.57	0	0.34
adipose tissue, adult, pool1 : CNhs10615 ctss [tpm]	0	5	1.52	0.15	0	0	0	0.15	0	0	0	0	0	0.91	0	0
aorta, adult, pool1 : CNhs11760 ctss [tpm]	0	1.16	0.5	0	0	0	0	0	0	0	0	0	0	0.83	0	0
appendix, adult : CNhs12842 ctss [tpm]	0	2.48	10.84	0	0	0	0	0	0	0	0	0	0	2.48	0	0
artery, adult : CNhs12843 ctss [tpm]	0	2.64	1.32	0	0	0	0	0	0	0	0	0	0	3.31	0	0
bladder, adult, pool1 : CNhs10616 ctss [tpm]	0	1.12	2.64	0	0	0	0	0	0	0	0	0	0	0.41	0	0
blood, adult, pool1 : CNhs11761 ctss [tpm]	0	0	0	0	0	0	0	0	0	0	0	0	0	6.74	0	0
bone marrow, adult : CNhs12845 ctss [tpm]	0.18	4.2	4.75	1.1	0	0	0.18	0.73	0	0.18	0.18	0	0.37	29.06	0	0.55
breast, adult, donor1 : CNhs11792 ctss [tpm]	0	5.87	5.13	0	0	0	0	0	0	0	0	0	0	2.2	0	0
cervix, adult, pool1 : CNhs10618 ctss [tpm]	0	1.24	1.74	0	0	0	0	0	0	0	0	0	0	1.16	0	0
colon, adult, pool1 : CNhs10619 ctss [tpm]	0	1.71	12.64	0	0	0	0	0	0	0	0	0.1	0	0.7	0	0
esophagus, adult, pool1 : CNhs10620 ctss [tpm]	0	1.46	1.27	0	0	0	0	0	0	0	0	0	0	0.36	0	0
heart, adult, pool1 : CNhs10621 ctss [tpm]	0	3.14	0.39	0	0	0	0	0	0	0	0	0	0	0	0	0
heart, fetal, pool1 : CNhs10653 ctss [tpm]	0	3.28	1.98	0	0	0	0	0	0	0	0	0	0	0.48	0	0
kidney, adult, pool1 : CNhs10622 ctss [tpm]	0	2.33	0.69	0	0	0	0	0	0	0	0	0	0	0.52	0	0
kidney, fetal, pool1 : CNhs10652 ctss [tpm]	0	2.18	1.09	0	0	0	0.07	0	0	0	0	0	0	0.58	0	0
liver, adult, pool1 : CNhs10624 ctss [tpm]	0	0.08	0.08	0	0	0	0	0	0	0	0	0	0	0.15	0	0
liver, fetal, pool1 : CNhs11798 ctss [tpm]	0	0	0	0	0	0	0	0	0	0	0	0	0	0	0	0
lung, adult, pool1 : CNhs10625 ctss [tpm]	0	0.1	0	0.1	0	0	0	0	0	0	0	0	0	0.21	0	0
lung, fetal, donor1 : CNhs11680 ctss [tpm]	0	0.63	0	0	0	0	0	0	0	0	0	0	0	0.47	0	0
lymph node, adult, donor1 : CNhs11788 ctss [tpm]	0	0.95	0.48	0	0	0	0	0	0	0	0.48	0	0	1.43	0	0
ovary, adult, pool1 : CNhs10626 ctss [tpm]	0	0.76	2.84	0	0	0	0	0	0	0.09	0	0	0	1.8	0	0
pancreas, adult, donor1 : CNhs11756 ctss [tpm]	0	0	0	0	0	0	0	0	0	0	0	0	0	0.5	0	0
placenta, adult, pool1 : CNhs10627 ctss [tpm]	0	6.33	8.47	0	0	0	0.19	0	0	0	0	0	0	2.14	0	0
prostate, adult, pool1 : CNhs10628 ctss [tpm]	0.1	13.25	82.22	0	0	0	0	0	0	0	0	0	0	0.3	0	0.1
skeletal muscle, adult, pool1 : CNhs10629 ctss [tpm]	0	1.41	0.94	0	0	0	0	0	0	0.08	0.08	0	0	0.31	0	0
skeletal muscle, fetal, donor1 : CNhs11776 ctss [tpm]	0	1.5	2.25	0	0	0	0	0	0	0	0	0	0	2.25	0	0
skin, adult, donor1 : CNhs11785 ctss [tpm]	0	0	0	0	0	0	0	0	0	0	0	0	0	0	0	0
skin, fetal, donor1 : CNhs11774 ctss [tpm]	0	1.24	0.41	0	0	0	0	0	0	0	0	0	0	0.83	0	0
small intestine, adult, pool1 : CNhs10630 ctss [tpm]	0	1.74	0.48	0	0	0	0.08	0	0	0	0	0	0	0.24	0	0
small intestine, fetal, donor1 : CNhs11773 ctss [tpm]	0	2.69	2.01	0	0	0	0	0	0	0	0	0	0	1.12	0	0
smooth muscle, adult, pool1 : CNhs11755 ctss [tpm]	0	0.92	1.15	0	0	0	0	0	0	0	0	0	0	0.69	0	0
spleen, adult, pool1 : CNhs10631 ctss [tpm]	0	0.34	0.11	0	0	0	0	0	0	0	0	0	0	9.63	0	0.23
spleen, fetal, pool1 : CNhs10651 ctss [tpm]	0	0.61	0.26	0	0	0	0	0	0	0	0	0	0	2.81	0	0
testis, adult, pool1 : CNhs10632 ctss [tpm]	0.15	1.2	2.02	0.07	0.07	0	0	0	0	0	0	0	0	4.26	0	0.07
testis, adult, pool2 : CNhs12998 ctss [tpm]	0.61	0.2	0.41	0	0	0	0	0	0	0	0	0	0	11.6	0.2	0
thymus, adult, pool1 : CNhs10633 ctss [tpm]	0	0.15	0.08	0	0	0	0	0	0	0	0	0	0	3.25	0	0
thymus, fetal, pool1 : CNhs10650 ctss [tpm]	0	0.23	0.16	0	0	0	0	0.08	0	0	0	0	0	5.75	0	0.08
thyroid, adult, pool1 : CNhs10634 ctss [tpm]	0	0.66	0.25	0	0	0	0	0	0	0	0	0	0	1.48	0	0
thyroid, fetal, donor1 : CNhs11769 ctss [tpm]	0	1.29	1.13	0	0	0	0.16	0	0	0	0	0	0	12.4	0	0
tongue, adult : CNhs12853 ctss [tpm]	0	1.79	0.65	0	0	0	0.16	0	0	0	0	0	0.16	0.49	0	0
tongue, fetal, donor1 : CNhs11768 ctss [tpm]	0	3.79	0.5	0	0	0	0	0	0	0	0	0.25	0	0.76	0	0
trachea, adult, pool1 : CNhs10635 ctss [tpm]	0	0.66	0.66	0	0	0	0	0	0	0	0	0	0	0.66	0	0
trachea, fetal, donor1 : CNhs11766 ctss [tpm]	0	1.52	2.12	0.3	0	0	0	0	0	0	0	0	0	0.3	0	0
umbilical cord, fetal, donor1 : CNhs11765 ctss [tpm]	0	3	16.48	0	0	0	0	0	0	0	0	0	0	8.99	0	0

Supplementary Figure S8. Expression of human mDA-ORs in selected human FANTOM5 hCAGE libraries. Color-coded representation of OR expression in human FANTOM5 hCAGE libraries. Values are expressed in tag per million (TPM). SN libraries are in red; tissue libraries that were validated by qRT-PCR are in blue.
