# Supplementary material for: Mesencephalic dopaminergic neurons express a repertoire of olfactory receptors and respond to odorant-like molecules
Source: BMC Genomics. 2014 Aug 27;15(1):729. doi: 10.1186/1471-2164-15-729 (PMC4161876; doi:10.1186/1471-2164-15-729)
Supplement: Supplementary file 11 — Additional file 11: Figure S9: Analysis of OR2L13 response to selected odors. Expression of human OR2L13 in HEK cells was verified by immunofluorescence (a) and western blotting (b) with anti-Rho antibody. pCDN3.1-empty vector and S6 OR expressing plasmid were used as negative and positive controls, respectively. For functional assays, OR2L13 was transiently transfected in HEK cells in combination with pCRE-SEAP. After transfection, cells were challenged with odor molecules at 600 μM concentration (c) or at the indicated quantities (d). Ringer’s solution or DMSO was used as control. mDA-OR activation was measured with fluorometric assay on culture medium. Data indicate mean ± st dev and are calculated on two independent experiments. (PDF 396 KB) [file 12864_2013_6425_MOESM11_ESM.pdf]

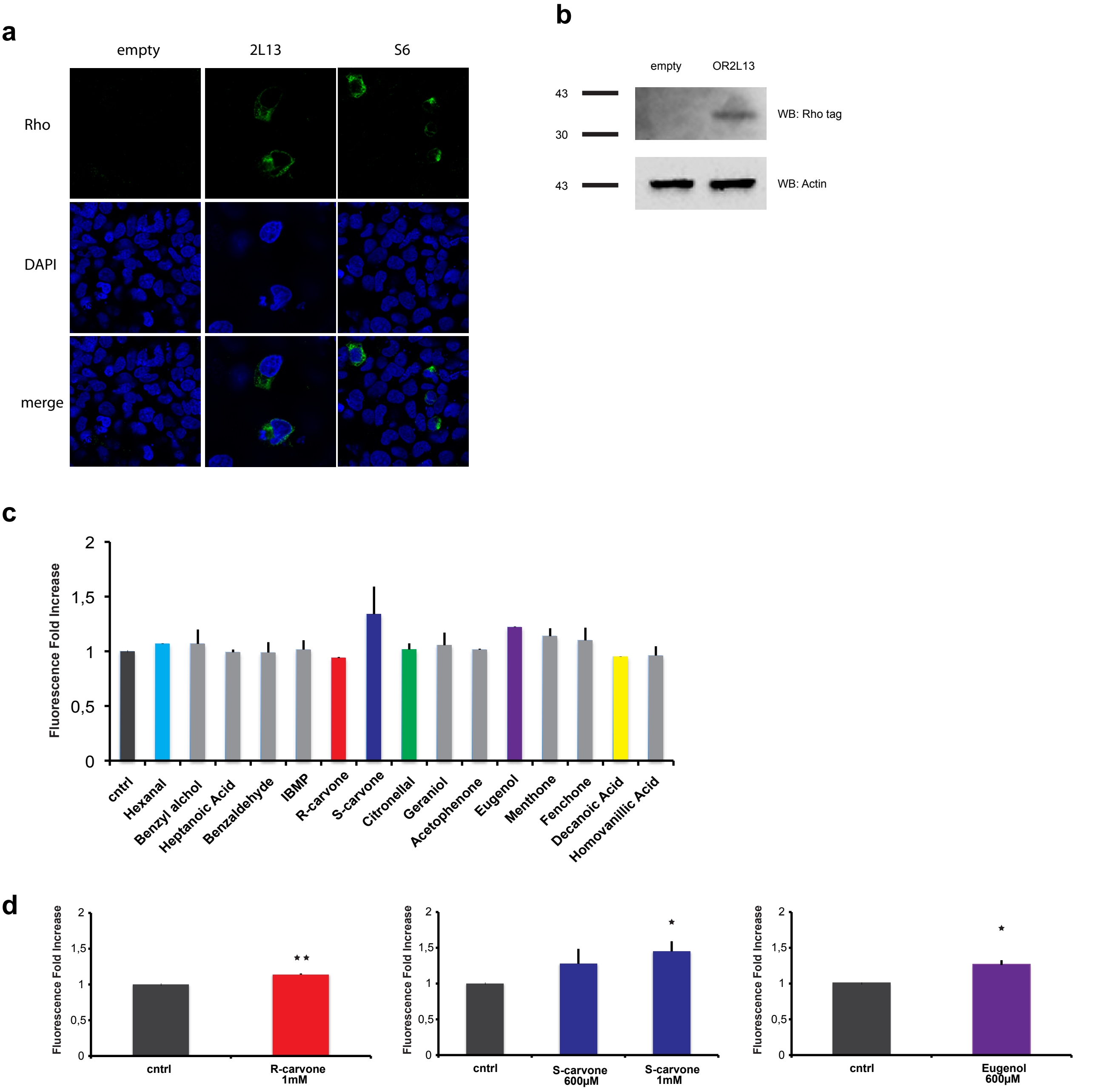

Supplementary Figure S9. Analysis of OR2L13 response to selected odors. Expression of human OR2L13 in HEK cells was verified by immunofluorescence **(a)** and western blotting **(b)** with anti-Rho antibody. pCDN3.1- empty vector and *S6* OR expressing plasmid were used as negative and positive controls, respectively. For functional assays, OR2L13 was transiently transfected in HEK cells in combination with pCRE-SEAP. After transfection, cells were challenged with odor molecules at 600μM concentration **(c)** or at the indicated quantities **(d)**. Ringer's solution or DMSO was used as control. mDA-OR activation was measured with fluorometric assay on culture medium. Data indicate mean ± st dev and are calculated on two independent experiments.
