# Supplementary material for: Mesencephalic dopaminergic neurons express a repertoire of olfactory receptors and respond to odorant-like molecules
Source: BMC Genomics. 2014 Aug 27;15(1):729. doi: 10.1186/1471-2164-15-729 (PMC4161876; doi:10.1186/1471-2164-15-729)
Supplement: Supplementary file 12 — Additional file 12: Figure S10: A subset of human Taste Receptors is regulated in PD. qRT-PCR of SN from control and PD post mortem brain samples. Data indicate mean ± stdev. *p < 0.05. (PDF 117 KB) [file 12864_2013_6425_MOESM12_ESM.pdf]

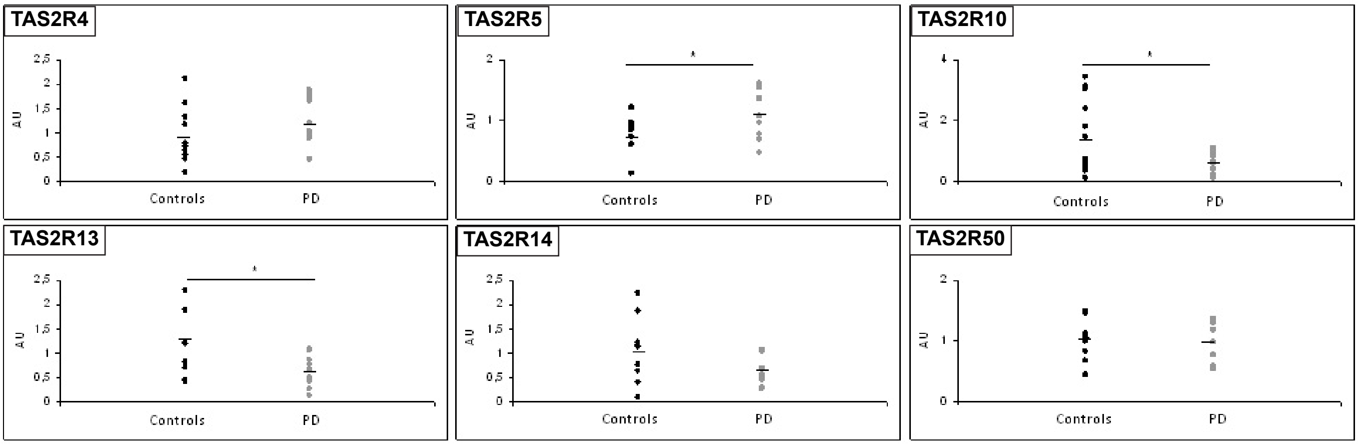

Supplementary Figure S10. A subset of human Taste Receptors is regulated in PD. qRT-PCR of SN from control and PD post-mortem brain samples. Data indicate mean ± stdev. * $p < 0.05$.
