# Supplementary material for: Mesencephalic dopaminergic neurons express a repertoire of olfactory receptors and respond to odorant-like molecules
Source: BMC Genomics. 2014 Aug 27;15(1):729. doi: 10.1186/1471-2164-15-729 (PMC4161876; doi:10.1186/1471-2164-15-729)
Supplement: Supplementary file 13 — Additional file 13: Table S3: List of primers. Complete list of oligonucleotides used in this study for non-quantitative PCR, quantitative RT-PCR, cloning and in situ hybridization. (PDF 54 KB) [file 12864_2013_6425_MOESM13_ESM.pdf]

Oligonucleotides for Non-quantitative PCR

Olfr78
fw: CTCACTTTTGGTTTGGCTTC
rev: AGCAGAATGGCAGTTAGACC
Olfr118
fw: TGTCATACCGAAGATGTTGC
rev: GTAGATGGGAGGAACAGGTG
Olfr1129
fw: CCACTGAGTGTTTTCTGCTG
rev: TGGGAGTCACAATGGTGTAA
Olfr130
fw: CAACTTGAGAAGATCCTCTTT
rev: CTGGATATAGATAGCAGTGAT
Olfr234
fw: TTCTTCCTCTGCCATTTCTC
rev: GAGAGTGCCGTAGAATACCG
Olfr287
fw: GGTCTTTCTAGTGGGTGTGG
rev: GGGGGTGATAATGGTGTAGA
Olfr299
fw: CTCAGGAATCTGTCTATTGTA
rev: AGATGATAGGATTTAAGAATG
Olfr316
fw: ATCATGGACATGATGTACATC
rev: ATCTGGATTACAACAACCAAC
Olfr420
fw: TCCCAAAGATGCTCTCTAGC
rev: AGGTAGCAGAGAAGCGAAGA
Olfr449
fw: CTTGCCAACCTGTCTTTCTT
rev: AAAAGGCTTTCTGCTTTCCT
Olfr502
fw: TGACATAGGCTATTCATCTTC
rev: GGACTTGGGCATCACATAAA
Olfr723
fw: ACATGTGTCTTTCCACTGTC
rev: GACCACAATTATGTGAGCAGA
Olfr883
fw: CTGCACAACCCCATGTACTA
rev: AGGATGTTGGAAAGGATCAA
Olfr885
fw: AATTTATCTTGCTGGGCTTG
rev: GGAGGTACTTGAGCAGGAGA
Olfr909
fw: TGGCCTATGATCGTTATGTG
rev: GGGCAATTTTAACATCCTTG
Olfr958
fw: CTGTGTCTCCCAGCTCTTCT
rev: GCATTGGACCTACCAAGTTC
Olfr1288
fw: AAGTCATTTCTTTCCACAGCT
rev: TGATGGCCAACCTCATATCTT
Olfr1344
fw: GATAGCCTTGGACAGACGAC
rev: CCAGGTGTAAGAGGCTAGGA
Olfr1443
fw: ACTGCAGACCCCACTCTTTA

rev: GGAGTGCAGAACAGATGACA
Olfr166
fw: CAAACAGGCCTGCTACTTTT
rev: CTTGTGCTCACAAACACCAT
Olfr1505
fw: ACGCTGGCTACAGGTAAAAC
rev: CAGGGATTTCTTTGAACCAC
Olfr1333
fw: TGGGTCTCTGAGTTCATCCT
rev: CAAGACCAAGTCCACCATCT
Olfr558
fw: TTCCCTTTGTGTTCCCTCTA
rev: GGCTGAGATGATGACAATGA
Olfr441
fw: TCAGACATGGATCACAGAGG
rev: ACAAGCTGCAAAGATGACAA

Oligonucleotides for quantitative RT-PCR

Olfr287
fw: AGTTCACCATGAAGTTGATCTTTGTG
rev:ATGTAAGAAAACATGGCTGAGGTGTAG
Olfr316
fw: GTGTGATCCAAGCCTTTGTTTTTCT
rev: CTAGAGCCACAGAATGGAAGTTGAA
Olfr558
fw: CTACCATCCAGTTTGATGCTTGTCT
rev: GAAGACAGGTAAGGGTGCCATTAGT
β-actin
fw: CACACCCGCCACCAGTTC
rev: CCCATTCCCACCATCACACC

Oligonucleotides for cloning

FL_olfr166
fw: ATGGAGAAATGGAATCAGAGCTCAAGTG
rev: TTACGGTTTAGTTGAAGGAAAAGTACCAAGGAC
FL+linker_olfr166
fw: ACTAGCGGCCGCCATGGAGAAATGGAATCAGAGCTCAAGTG
rev:ACTAGAATTCTTACGGTTTAGTTGAAGGAAAAGTACCAAGGAC

FL_olfr287
fw: ATGAGGTCCCAGACAGCAGAC
rev: CTAGACAGACTCTGCCTGAGCCA
FL+linker_olfr287
fw: ACTAGCGGCCGCCATGAGGTCCCAGACAGCAGAC
rev: ACTAGCGGCCGCCATGAGGTCCCAGACAGCAGAC

FL_olfr316
fw: ATGGAGTCAGGAAACCGCAGC
rev: TCATTTCATTTGTCTCCTCAGGGCCC
FL+linker_olfr316
fw: ACTAGCGGCCGCCATGGAGTCAGGAAACCGCAGC
rev: ACTAGAATTCTCATTTCATTTGTCTCCTCAGGGCCC

FL_olfr558
fw: ATGGTGGGCTTCAATAGCAATGAATCC
rev: CTAGTGATCTGAAGTGTGTGTGGTCACG

FL+linker_olfr558
fw: ACTAGCGGCCGCCATGGTGGGCTTCAATAGCAATGAATCC
rev: ACTAGAATTCCTAGTGATCTGAAGTGTGTGTGGTCACG

FL_olfr883
fw: ATGGTATTAGAAAATTCCTCTTCAGTG
rev: TTAGGTAAAACTCCTTTTCATCAAAGT
FL+linker_olfr883
fw: ACTAGCGGCCGCCATGGTATTAGAAAA
rev: ACTAGAATTCTTAGGTAAAACTCCTTTTCATCAAAGT

FL_olfr1344
fw: ATGGGCCCGGGAACCCTCAAT
rev: TTAGTGCAACACAATGCGCGTCCC
FL+linker_olfr1344
fw: ACTAGCGGCCGCCATGGGCCCGGGAACCCTCAAT
rev: ACTAGAATTCTTAGTGCAACACAATGCGCGTCCC

Oligonucleotides for ISH probe preparation

Olfr287
fw: GGTCTTTCTAGTGGGTGTGG
rev: GGGGGTGATAATGGTGTAGA
Olfr316
fw: ATCATGGACATGATGTACATC
rev: ATCTGGATTACAACAACCAAC
Olfr558
fw: CCAGGATTGGAAGAGGTTCA
rev: CTCCATGCCAGACAAGGAAT

Supplementary Table 3. List of primers. Complete list of oligonucleotides used in this study for non-quantitative PCR, quantitative RT-PCR, cloning and in situ hybridization.
